# Dopamine, sleep, and neuronal excitability modulate amyloid-β–mediated forgetting in *Drosophila*

**DOI:** 10.1371/journal.pbio.3001412

**Published:** 2021-10-06

**Authors:** Jenifer C. Kaldun, Shahnaz R. Lone, Ana M. Humbert Camps, Cornelia Fritsch, Yves F. Widmer, Jens V. Stein, Seth M. Tomchik, Simon G. Sprecher

**Affiliations:** 1 Department of Biology, University of Fribourg, Fribourg, Switzerland; 2 Department of Animal Sciences, Central University of Punjab, Bathinda, India; 3 Department of Medicine, University of Fribourg, Fribourg, Switzerland; 4 Department of Neuroscience, The Scripps Research Institute, Jupiter, Florida, United States of America; Stony Brook University Medical Center: Stony Brook University Hospital, UNITED STATES

## Abstract

Alzheimer disease (AD) is one of the main causes of age-related dementia and neurodegeneration. However, the onset of the disease and the mechanisms causing cognitive defects are not well understood. Aggregation of amyloidogenic peptides is a pathological hallmark of AD and is assumed to be a central component of the molecular disease pathways. Pan-neuronal expression of *Aβ*_*42*_^*Arctic*^ peptides in *Drosophila melanogaster* results in learning and memory defects. Surprisingly, targeted expression to the mushroom bodies, a center for olfactory memories in the fly brain, does not interfere with learning but accelerates forgetting. We show here that reducing neuronal excitability either by feeding Levetiracetam or silencing of neurons in the involved circuitry ameliorates the phenotype. Furthermore, inhibition of the Rac-regulated forgetting pathway could rescue the *Aβ*_*42*_^*Arctic*^-mediated accelerated forgetting phenotype. Similar effects are achieved by increasing sleep, a critical regulator of neuronal homeostasis. Our results provide a functional framework connecting forgetting signaling and sleep, which are critical for regulating neuronal excitability and homeostasis and are therefore a promising mechanism to modulate forgetting caused by toxic *Aβ* peptides.

## Abstract 2

One of the early hallmarks in Alzheimer’s disease is increased forgetting. This study shows that restricted expression of amyloid beta in the memory center of fruit flies causes enhanced forgetting without affecting the ability to learn; forgetting is caused by increased excitability and can be restored with drugs, increased sleep or modulation of dopamine signalling.

## Introduction

Alzheimer disease (AD) is an age-related neurodegenerative disease and the most common cause of dementia in elderly people [1–3]. The prevalence of the disease is predicted to rise within the next decades, making a huge impact on healthcare systems and individuals [3,4]. Characteristic symptoms are a progressive decline in cognitive functions, gradual memory loss, and impairment of locomotor functions [1–4]. Moreover, changes in sleeping patterns and neuronal firing are widely observed among patients [3,5,6]. Hallmarks of AD include the intercellular accumulation of β-amyloid (Aβ) plaques, intracellular neurofibrillary tangles (NFTs) made of abnormal Tau protein, and loss of neurons [7,8]. It is believed that the initiation of AD happens years before the first symptoms are evident [9,10]. In this preclinical AD phase, the brain might be able to compensate for the neuronal changes upon Aβ accumulation, therefore limiting behavioral and cognitive symptoms. However, the early steps of the disease and the role of Tau and Aβ are not well understood [11]. Hereditary, early-onset cases of AD carry mutations in the cleavage process of the amyloid beta precursor protein (APP). APP is a transmembrane protein that seems to play a role in synaptic regulations and neuronal survival. It is cleaved by 3 secretases (α-, β-, and γ-secretase) into various intra- and extracellular fragments [12,13]. Sequential cleavage by β- and γ-secretase produces the 42 amino acids long Aβ_42_, a peptide with high aggregation potential and the main compound of the Aβ plaques [2,3,8,14,15]. However, it remains elusive how Aβ_42_ aggregation causes neuronal dysfunction [14,16,17].

Increasing the Aβ_42_ amount by distinct methods in animal models recaptures AD phenotypes [18]. This further highlights the role of Aβ_42_ in the disease and suggests that animal models can be used to better understand the basic mechanisms of AD.

The fruit fly *Drosophila melanogaster* is a widely used model organism for neurobiological diseases including AD, offering versatile genetic tools [19]. Several models have been developed to assess the molecular and genetic aspects of AD [20–25]. Pan-neuronal expression of amyloidogenic Aβ_42_ peptides in the fly causes progressive locomotor deficits, reduced life span, and progressive learning defects [23,24,26–28].

The Arctic variant (E22G) Aβ_42_ peptide (*Aβ*^*Arctic*^), occurring in familial AD cases, is more prone to aggregation than wild-type Aβ_42_ and thus has a faster and more severe phenotype [16,28]. In the current study, we first showed that the expression of *Aβ*^*Arctic*^, using pan-neuronal drivers, causes defects in 0-h and 2-h memory. However, restricted expression of *Aβ*^*Arctic*^ to the mushroom bodies (MBs), a brain center involved in olfactory learning, did not alter 0-h memory but caused a reduction of memory performance at 2 h and later time points. This accelerated forgetting of 2-h memory was restored by using the anticonvulsant drug Levetiracetam (LEV) or by silencing the MB neurons expressing *Aβ*^*Arctic*^. Further, by temporally silencing neurons of the forgetting circuitry in flies expressing *Aβ*^*Arctic*^ in the MB, we were able to rescue memory defects. A similar rescue was obtained by blocking the forgetting pathway. Moreover, inducing sleep by artificial means (either by pharmacological or by genetic manipulations) restored memory defects. Thus, our results provide experimental evidence that signals caused by toxic Aβ peptides, which are responsible for memory decay, are linked to the forgetting circuitry by yet unknown mechanisms. These observations point to a more general mechanism linking Aβ to forgetting, sleep, and neuronal excitability.

## Results

### Restricted amyloid β expression accelerates short-term memory forgetting

**Fig 1:**
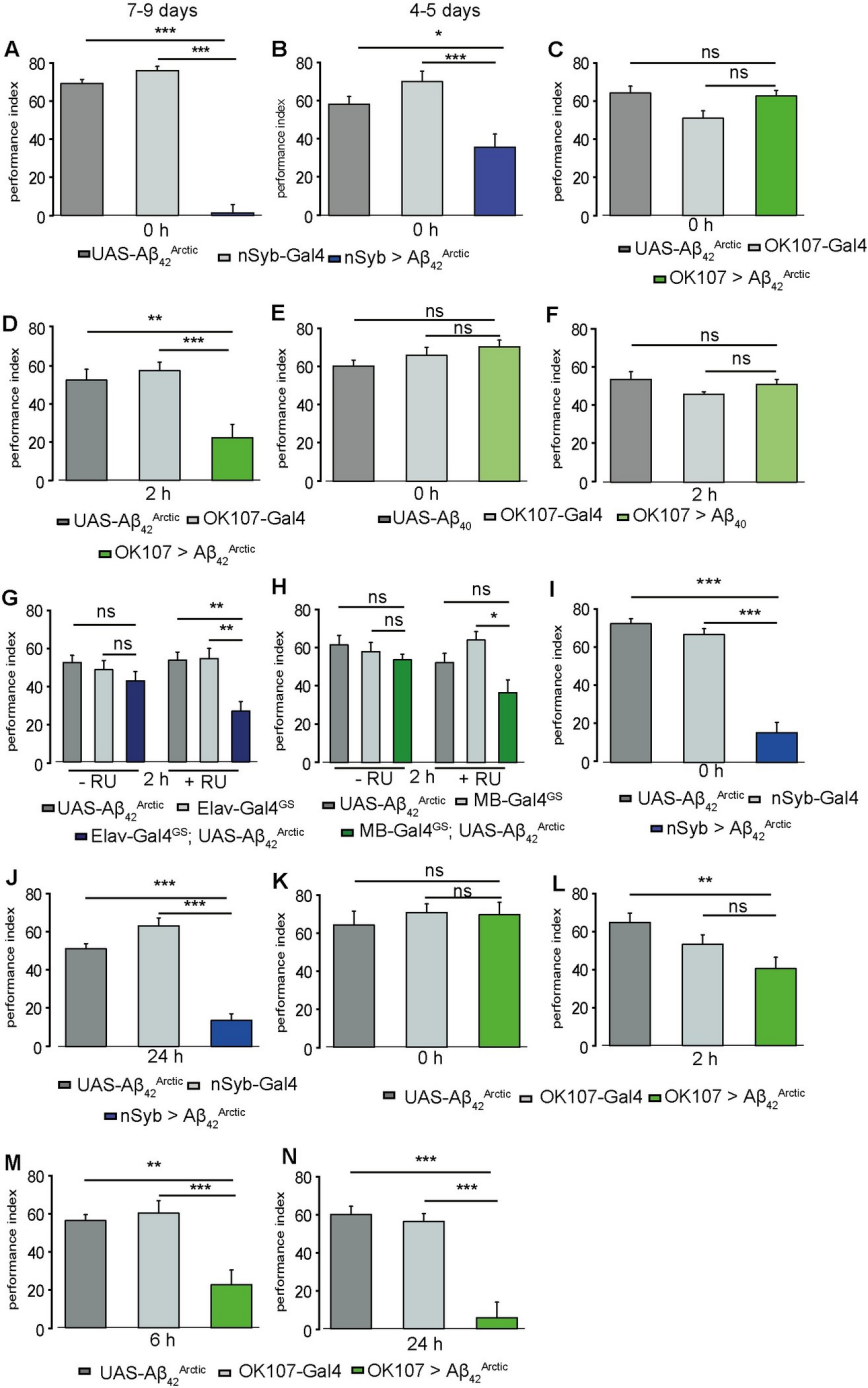
Expression of amyloid β peptides in the brain or MBs leads to memory defects in aversive and appetitive conditioning. (A-H) Aversive olfactory conditioning experiments were conducted with *Aβ*_*42*_^*Arctic*^ expressing flies. (A, B) Pan-neuronal expression of *Aβ*_*42*_^*Arctic*^ impaired memory measured immediately (0 h) after training in 7- to 9-day-old flies (A) 4- to 5-day-old *nSyb>Aβ*_*42*_^*Arctic*^ flies (B), n ≥ 12. (C, D) Flies expressing *Aβ*_*42*_^*Arctic*^ in the MB using *Ok107-Gal4* did not perform differently from parental controls for 0-h memory (C; n ≥ 12) but showed a significantly lower 2-h memory performance (D; n ≥ 12). (E, F) Flies expressing the nontoxic *Aβ*_*40*_ in the MB have no significant memory impairment 0 h (E) or 2 h (F) after conditioning (n ≥ 12). (G, H) The GeneSwitch Gal4 system was used to restrict the expression of *Aβ*_*42*_^*Arctic*^ to the adult stage, by feeding flies RU486 after the hatching for 8 days until the conditioning experiments. (G) *elav*-*Gal4*^*GS*^; *Aβ*_*42*_^*Arctic*^ flies fed with RU486 showed a lower performance index than vehicle-treated flies 2 h after training (n ≥ 12). (H) Induction of *Aβ*_*42*_^*Arctic*^ expression after hatching in MB cells using *MB*-*Gal4*^*GS*^ resulted in a slightly reduced 2-h memory performance (n ≥ 12). (I-N) Appetitive olfactory conditioning experiments were performed with flies expressing *Aβ*_*42*_^*Arctic*^ in the brain or specifically in the MB. (I, J) Expression of *Aβ*_*42*_^*Arctic*^ in the whole brain impaired memory measured immediately (I; n ≥ 12) and 24 h after conditioning (J; n ≥ 12). (K-N) MB-specific expression of *Aβ*_*42*_^*Arctic*^ using *OK107-Gal4* did not affect 0-h memory (K), whereas 2-h (L), 6-h (M), and 24-h (N) memory performances were reduced in AD flies compared to parental control flies (n ≥ 12). See S1–S3 Figs for sensory controls and S1 Table for the data. Bar graphs represent the mean, and error bars represent the standard error of the mean. Asterisks denote significant difference between groups (*p < 0.05, **p < 0.005, ***p < 0.001). AD, Alzheimer disease; MB, mushroom body.

The pan-neuronal expression of Aβ_42_ peptides in the nervous system of *D*. *melanogaster* causes AD phenotypes and symptoms like those observed in other animal models and patients. Among them are neurodegeneration, the formation of plaque-like accumulations, a reduced life span, impaired locomotor ability, disruptions in sleep, and in circadian rhythms. Pan-neuronal expression of Aβ_42_ peptides is also reported to interfere with the ability of the fly to learn and form memories [26–28]. Before testing memory, we first tested if the *UAS-Aβ*_*42*_^*Arctic*^*; nSyb-Gal4* (*nSyb*>*Aβ*_*42*_^*Arctic*^) flies are capable of sensing the used stimuli. The response to both odors—4-methyl-cyclohexanol (MCH) and 3-Octanol (3-Oct)—is comparable to the parental control lines (S1A and S1B Fig). Furthermore, the response to electric shock is similar to the controls (S1C Fig). In aversive olfactory conditioning, using electric shocks as reinforcement, we observed that 7- to 9-day-old *nSyb*>*Aβ*_*42*_^*Arctic*^ flies showed a reduced short-term memory (STM) performance compared to parental controls (Fig 1A). In agreement with previous studies [26,28], we observed that the pan-neuronal expression of *UAS-Aβ*_*42*_^*Arctic*^ with *nSyb-Gal4* causes reduced locomotion/mobility and reduced viability (S1D Fig). Therefore, to avoid a negative impact by reduced mobility, we did the same experiment with 4- to 5-day-old flies. Similar to the older flies, these *nSyb*>*Aβ*_*42*_^*Arctic*^ flies also showed a significant learning defect (Fig 1B). Therefore, we decided to use younger *nSyb*>*Aβ*_*42*_^*Arctic*^ flies to circumvent problems with reduced mobility. Furthermore, 2 h after conditioning, *nSyb*>*Aβ*_*42*_^*Arctic*^ flies performed worse than control lines (S1D Fig). As *nSyb*>*Aβ*_*42*_^*Arctic*^ flies have normal responses to the used odors as well as to electric shock, Aβ seems to interfere with learning itself rather than with stimulus detection. Our observations fit with previous reports suggesting that pan-neuronal Aβ expression shows STM deficits [26–28].

To test for spatially restricted deficits caused by amyloidogenic Aβ peptides, we expressed *Aβ*_*42*_^*Arctic*^ specifically in the MB, the main center for learning and storing of olfactory memories [29–31]. We verified the expression using an *Aβ*_*42*_-specific monoclonal antibody and a recently published protocol [32]. Compared to the wt line, the *Ok107>Aβ*_*42*_^*Arctic*^ brains show a clear signal around the area of the MB (S7A and S7B Fig). Flies expressing *Aβ*_*42*_^*Arctic*^ restricted to the MB using OK107-Gal4 (*OK107>Aβ*_*42*_^*Arctic*^) showed normal scores in the sensory tests at age 7 to 9 days speaking against interference with stimuli detection (S1E–S1G Fig). Astonishingly, we found that 7- to 9-day-old OK107>*Aβ*_*42*_^*Arctic*^ flies showed a normal learning performance immediately after training. The performance index was not significantly different from the parental control strains (Fig 1C). However, when testing memory performance 2 h after training, we found that *OK107>Aβ*_*42*_^*Arctic*^ flies showed a lower performance compared to the controls (Fig 1D). As the performance index of *OK107>Aβ*_*42*_^*Arctic*^ is not significantly different from the controls at 0 h, we assume that flies expressing *Aβ*_*42*_^*Arctic*^ in the MB can learn normally but have a faster forgetting rate. However, we cannot exclude that those flies have an impaired memory consolidation. A recent study in a mouse AD model observed a forgetting phenotype without a learning defect in young mice and termed it accelerated forgetting [33]. Thus, we will use accelerated forgetting to describe our phenotype. We also observe this accelerated forgetting 4 and 6 h after training (S1H Fig shows the memory retention at 0 h, 2 h, 4 h, and 6 h after aversive training). Expression of the nontoxic *Aβ*_*40*_ variant in the MB does not affect learning at either 0 h or 2 h after training (Fig 1E and 1F), indicating that the observed effect is based on *Aβ*_*42*_^*Arctic*^ itself.

Two components of aversive memory can be distinguished in *Drosophila*, anesthesia-sensitive memory (ASM) and anesthesia-resistant memory (ARM) [34]. To examine if ARM is affected by expressing *Aβ*_*42*_^*Arctic*^ in the MB, flies were subjected to a cold shock after conditioning that eliminates the labile ASM component and the 2 h memory was tested. The reduced 2-h memory performance of *Aβ*_*42*_^*Arctic*^ expressing flies in the MB was further decreased by cold shock treatment, indicating that also ARM components might have a higher forgetting rate (S1I Fig).

In order to exclude developmental defects, we restricted Gal4 activity to the adult stage using the GeneSwitch GAL4 system, which allows the activation of a chimeric GAL4 protein by feeding flies the steroid Mifepristone (RU486) [35]. Adult flies were aged for 7 days on food containing 250 μM RU486 or only solvent. Adult-specific pan-neuronal expression of *Aβ*_*42*_^*Arctic*^ using an *elav-Gal4*^*GS*^ driver did not alter sensory perception (S2A–S2C Fig). Surprisingly, immediate memory after aversive olfactory conditioning was comparable to parental control lines (S2D Fig). However, we observed accelerated forgetting 2 h after training when compared to animals that did not receive RU486 (Fig 1G). Similar results were also obtained with an adult-specific expression of *Aβ*_*42*_^*Arctic*^ restricted to the MB using an *MB-Gal4*^*GS*^ driver at both 0 h (S2E Fig) and 2 h (Fig 1H) after training. However, the phenotype was much milder, arguing again for an effect of amount or duration *Aβ*_*42*_^*Arctic*^ accumulation.

Given that *Aβ*_*42*_^*Arctic*^ accumulation seems to correlate with severity of memory impairment, we tested younger (4 to 5 days) and older (14 to 15 days) *OK107>Aβ*_*42*_^*Arctic*^ flies. Younger *OK107>Aβ*_*42*_^*Arctic*^ flies showed normal performance scores 2 h after aversive training (S2F Fig). It seems to require a few days of *Aβ*_*42*_^*Arctic*^-expression to obtain accelerated forgetting. In contrast, older *OK107>Aβ*_*42*_^*Arctic*^ flies showed a memory impairment directly after training (S2G Fig).

In summary, we observe that temporally and spatially restricted *Aβ*_*42*_^*Arctic*^ expression results in accelerated forgetting. This could be due to impaired consolidation or a more active forgetting pathway. Moreover, the observation that adult-specific pan-neuronal expression of *Aβ*_*42*_^*Arctic*^ does not cause learning defects suggests that reduced associative memory might be due to developmental defects caused by *Aβ*_*42*_^*Arctic*^ expression. Another explanation would be higher amounts of *Aβ* peptides accumulating in more brain areas. Nevertheless, the learning defects could also be caused by neurons included in the *nSyb-Gal4* driver line but not in the *OK107-Gal4* line. These findings suggest that the restricted expression of *Aβ* peptides might resemble earlier stages of AD.

### Long-term memory is affected by the expression of Aβ_42_^Arctic^

We were interested to test if *Aβ*_*42*_^*Arctic*^ is also affecting reward learning and long-term memory (LTM), by using sucrose as a reward in an olfactory conditioning paradigm. A single trial of appetitive olfactory conditioning is sufficient to induce LTMs [36,37]. Flies expressing *Aβ*_*42*_^*Arctic*^ pan-neuronally using *nSyb-Gal* or restricted to the MB with *OK107-Gal4* showed normal sugar response (S3A and S3B Fig). Like for aversive STM, 4- to 5-day-old *nSyb*>*Aβ*_*42*_^*Arctic*^ flies showed a reduced memory performance after appetitive training (Fig 1I). This further confirms that unrestricted *Aβ*_*42*_^*Arctic*^ expression throughout development or in more neuronal cells does interfere with memory acquisition. Because appetitive LTM forms in parallel with appetitive STM [38], we wanted to test if both memory phases are affected. *NSyb-Aβ*_*42*_^*Arctic*^ expressing flies also displayed a drastically impaired LTM performance, which was evaluated by testing the flies 24 h after conditioning (Fig 1J). Thus, both memory phases are affected by *Aβ*_*42*_^*Arctic*^. We next assessed if accelerated forgetting by the targeted expression of *Aβ*_*42*_^*Arctic*^ to the MB also affects reward middle term memory (MTM) and LTM. The flies were tested 0 h, 2 h, 6 h, or 24 h after training. Comparable to aversive training, we found that *OK107>Aβ*_*42*_^*Arctic*^ flies do not show differences in learning compared to parental control strains when tested immediately after training (Fig 1K). At 2 h after training, the *OK107>Aβ*_*42*_^*Arctic*^ flies seem to have a lower memory score than the control flies; however, the difference is not statistically significant. But at 6 h, and 24 h after training, *OK107>Aβ*_*42*_^*Arctic*^ flies showed accelerated forgetting (Fig 1L-1N). Thus, spatially restricted expression of *Aβ*_*42*_^*Arctic*^ to the MB does not interfere with the capability of the fly to form memories but results in more rapid memory decay and forgetting.

### Restricted Aβ expression to dopaminergic neurons

Previous studies [26–28], as well as this study, show that pan-neuronal expression of *Aβ* causes learning defects. As the expression of *Aβ* in the MB, the main learning center does not affect learning, these defects could be due to other neurons in the *nSyb* driver line required for learning. One candidate would be the dopaminergic neurons (DANs), which have a well-established role in learning [39,40]. Therefore, we expressed *Aβ*_*42*_^*Arctic*^ in the DANs using the *TH-Gal4* driver comprising most of the DANs [41,42]. The 0-h aversive memory of those *TH*-*Aβ*_*42*_^*Arctic*^ flies was significantly reduced compared to the parental lines (S4A Fig). The PAM DANs required for reward learning are not well covered in the *TH-Gal4* line [39,43], so we used *GMR58E02-Gal4* (*PAM-Gal4*). Appetitive memory tested directly after training is significantly lower in *PAM-Aβ*_*42*_^*Arctic*^ flies compared to the parental controls (S4B Fig).

All in all, expression of *Aβ*_*42*_^*Arctic*^ in DANs seems to interfere with learning. Compared with *nSyb-Aβ*_*42*_^*Arctic*^ flies of the same age, the learning defect seems less severe.

### Altering neuronal excitability decreases Aβ-mediated forgetting

**Fig 2:**
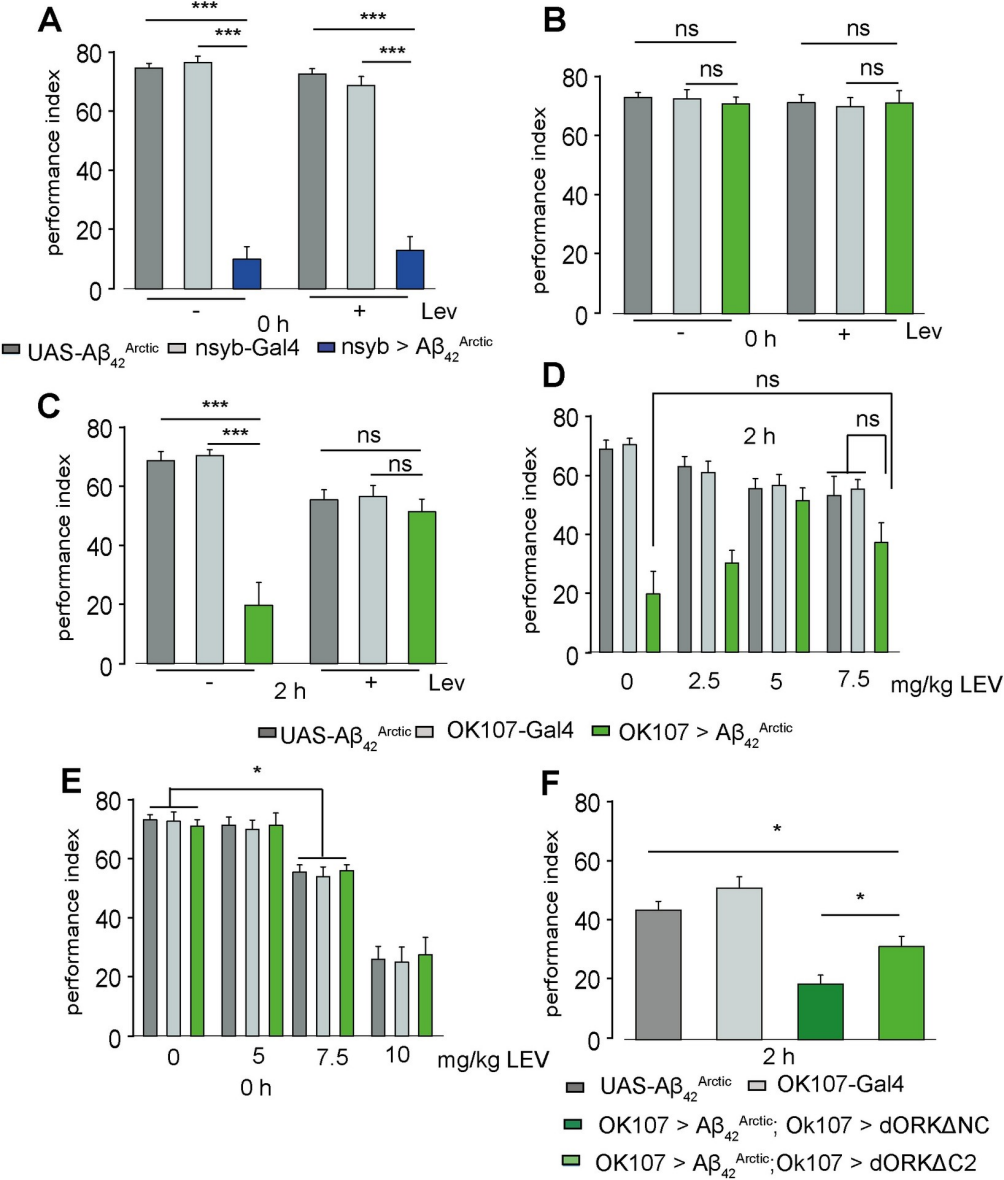
Reducing neuronal excitability with LEV or dORKΔC rescues memory defects caused by the expression of *Aβ_42_^Arctic^*. (A) Pan-neuronal expression of *Aβ*_*42*_^*Arctic*^ reduced aversive 0-h memory, which could not be restored with LEV treatment (n ≥ 12). (B) LEV treatment does not affect flies expressing *Aβ*_*42*_^*Arctic*^ in the MB directly after training (n ≥ 12) but (C) restored the impaired 2-h memory performance in flies expressing *Aβ*_*42*_^*Arctic*^ in the MB (n ≥ 12). (D) Flies were fed with either 2.5 or 7.5 mg/kg LEV, and 2-h aversive memory was tested. The data for 5 mg/kg are from Fig 3C (n ≥ 12). Other LEV concentrations do not seem to improve the memory performance. (E) Flies were fed with either 7.5 or 10 mg/kg LEV, and 0-h aversive memory was tested. The data for 5 mg/kg are from Fig 3B (n ≥ 12). Higher LEV concentrations seem to have a negative effect on the flies’ ability to learn (F) Expression of the nonconducting K^+^-channel dORKΔNC or constitutive conducting dORKΔC in the MB of *Aβ*_*42*_^*Arctic*^ -expressing flies. At 2 h after training, dORKΔC is able to partially restore the learning performance compared to dORKΔC (n ≥ 12). See S4 Fig for the further data on LEV and dORK and S1 Table for the data. All other details are similar to Fig 1. LEV, Levetiracetam; MB, mushroom body.

Brain imaging studies in AD patients show that neurons are hypoexcitable. However, patients with mild cognitive impairment (MCI), a pre-stage of AD, show hyperexcitability [2,6,44]. Therefore, the excitability of neurons seems to play a role in the disease progression with hyperexcitability occurring in early stages and hypoexcitability in later stages. Giving that restricting the expression of *Aβ*_*42*_^*Arctic*^ to the MB might represent an early stage of AD, we hypothesized that increased neuronal activity could lead to accelerated forgetting. Moreover, a recent study showed that flies expressing *Aβ*_*42*_ in the MB have an enhanced firing rate when monitoring individual neurons in an ex vivo preparation [45], indicating a similar mechanism in the fly. Previous studies in mammals and flies have shown that the anticonvulsant drug LEV improves *Aβ*-mediated phenotypes [46–51]. LEV could rescue the neuronal firing frequency in rodent and human MCI patients [46–49,51] In addition, in flies, it also seems to prolong the shortened life span of pan-neuronal *Aβ* expression. Moreover, the flies had a decreased firing rate [50]. Furthermore, LEV has been shown to improve learning and memory in rodent AD models [46–51]. Therefore, we assessed if LEV administration may also influence *Aβ*-mediated forgetting by feeding flies for 7 days with 5 mg/kg LEV and then testing their behavior. This concentration was previously used in a *Drosophila* AD model and showed a reduced spontaneous firing rate [50]. Pan-neuronal expression of *Aβ*_*42*_^*Arctic*^ decreases the learning performance directly after training. This learning defect could not be rescued by LEV feeding, as no difference in 0-h memory performance was observed between animals that were fed with LEV and animals that were fed with control food (Fig 2A). Furthermore, 2 h after training, there seems to be no rescue (S5A Fig). Thus, LEV cannot ameliorate the defects caused by developmentally expressed *Aβ*_*42*_^*Arctic*^, at least in our tested conditions.

Next, we asked whether administration of LEV can rescue the accelerated forgetting defect in flies where *Aβ*_*42*_^*Arctic*^ peptides are specifically targeted to the MB. We found that feeding 5 mg/kg LEV to *OK107>Aβ*_*42*_^*Arctic*^ did not affect memory formation (Fig 2B), but significantly improved the 2-h memory of AD flies, resulting in a performance index comparable to parental control lines (Fig 2C). These experiments indicate that LEV feeding could overcome the *Aβ*_*42*_^*Arctic*^-mediated forgetting, maybe by decreasing neuronal excitability. However, the effects of LEV on organisms are not well resolved, and the drug might have broad effects on brain functions. An important player in regulating neuronal homeostasis is sleep. To rule out that the observed effect is due to altered sleep, we monitored the sleep of flies fed with different concentrations of LEV, (1, 2, 3, 4, and 5 mg/kg) (S5B Fig). We did not find a major effect of LEV on sleep, especially in the used concentration of 5 mg/kg. Feeding LEV does not seem to change the expression of *Aβ*_*42*_^*Arctic*^ in the MB (S7A and S7B Fig).

Although 5 mg/kg seems to work well for us, we tested further LEV concentration and their effect on accelerated forgetting and learning. First, we tested if a lower concentration, 2.5 mg/kg, is able to rescue the 2-h memory of *OK107>Aβ*_*42*_^*Arctic*^ flies. Albeit the 2.5 mg/kg–fed *OK107>Aβ*_*42*_^*Arctic*^ flies have a slightly higher performance score than the vehicle fed ones, the improvement is not significant (Fig 2D). We also tested 7.5 mg/kg as a higher concentration. Compared to parental flies fed with 7.5 mg/kg, LEV-fed *OK107>Aβ*_*42*_^*Arctic*^ flies did not perform significantly differently. However, compared to vehicle-fed flies, the memory performance was not significantly different (Fig 2D). We presumed that this concentration might have side effects and tested the learning capability of the flies. In comparison to vehicle-fed flies, the 7.5-mg/kg flies have a slight learning defect independent of genotype. We further tested 10 mg/kg and saw a severe learning defect in all tested genotypes (Fig 2E). We thus conclude that LEV works doses dependent on the flies and our model. Too low concentrations are not sufficient to rescue the accelerated forgetting phenotype, whereas too high concentrations are detrimental. However, adapting the feeding duration, for example, feeding the lower concentration for more days, could provide the desired effect. *Aβ*_*42*_^*Arctic*^ in the MB might change the excitability of the MB intrinsic neurons causing a higher forgetting rate. To address this, we expressed *dORK*, a rectifying K^+^-Channel, to silence the MB neurons. The truncated *dORKΔC2*-construct is constantly open, whereas the *dORKΔNC2*-construct is not conducting [52]. Previous, *dORKΔC2* has been shown to reduce the neuronal firing but does not completely silence the neurons. The *dORKΔNC* construct does not affect neuronal firing [52]. Therefore, only the *dORKΔC2* construct should affect our phenotype. At 0 h after training, both *dORKΔC2*- and *dORKΔNC*-expressing flies showed normal learning scores (S5C Fig). As expected, expressing the d*ORK* constructs alone in the MB did not affect learning per se (S5D Fig). At 2 h after training, flies expressing the nonconducting *dORKΔNC* showed *Aβ*^*Arctic*^-mediated accelerated forgetting (Fig 2F). In contrast, the *dORKΔC2* flies showed improved memory scores 2 h after training. At this time point, the performance of the constructs alone is not too different from normal learning scores (S5E Fig).

Taken together, it seems that silencing the MB neurons can rescue the accelerated forgetting mediated by *Aβ*_*42*_^*Arctic*^. This implies that the *Aβ*_*42*_^*Arctic*^ peptide in flies could be influencing neuronal activity and therefore appears to act in a similar fashion as in mammals.

### Silencing dopaminergic forgetting neurons restores Aβ-mediated forgetting

**Fig 3:**
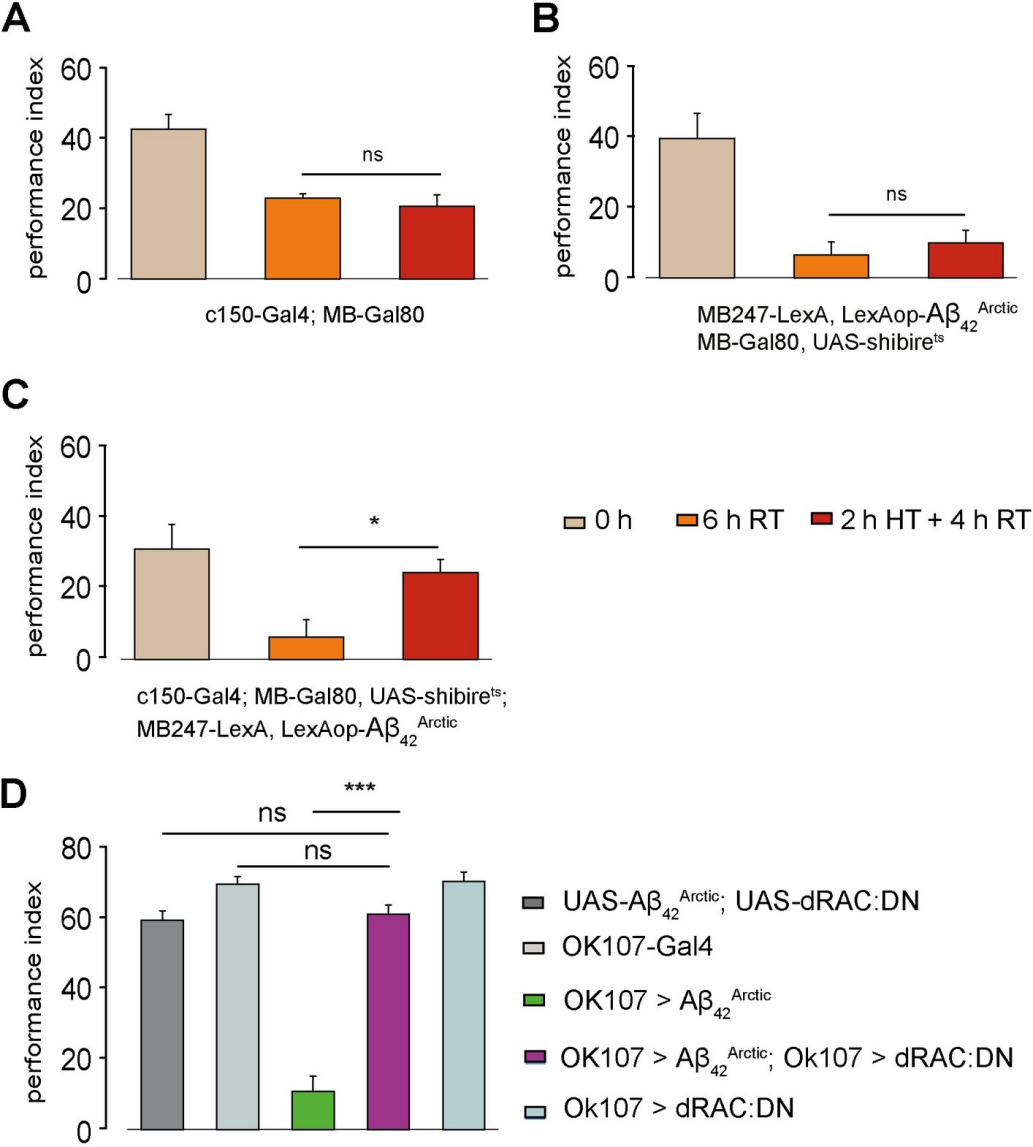
Inhibition of a set of DANs or the forgetting pathway improves memory defects caused by the expression of *Aβ_42_^Arctic^*. (A-C) Flies were conditioned with the appetitive olfactory paradigm and tested immediately afterward (0 h, depicted in buff), after 6 h at RT (depicted in orange) or after 2 h at HT (30°C) followed by 4 h at RT (depicted in red). (A) Memory performance of the *c150-Gal4*; *MB-Gal80* driver line that labels PPL1 DANs (n ≥ 8). (B) The HT regime did not significantly alter 6-h memory performance in flies expressing *Aβ*_*42*_^*Arctic*^ in the MB (n ≥ 8). (C) Inhibiting PPL1 neurons in the first 2 h after memory formation enhanced 6-h memory in *Aβ*_*42*_^*Arctic*^ expressing flies (n ≥ 8). (D) Expression of a dominant-negative dRac allele in the MB together with *Aβ*^*Arctic*^ (purple) is able to rescue the *Aβ*_*42*_^*Arctic*^ induced enhance forgetting phenotype (green) to wild-type levels. S1 Table includes the data. All other details are similar to Fig 1. DAN, dopaminergic neuron; HT, high temperature; MB, mushroom body; RT, room temperature.

Previous studies showed that forgetting is an active, tightly regulated biological process, which may be modulated by specific neuronal circuits. A defined set of DANs from the PPL1 cluster was shown to be critical for active forgetting and opposing memory consolidation [53–57]. Genetically silencing the MP1, MV1, and V1 DANs of the PPL1 cluster using the *c150-Gal4* driver line results in reduced forgetting and thus higher memory performance [54]. Moreover, this forgetting circuitry is downstream of the MB and gets activated after learning. Ongoing oscillations from DANs are required for the consolidation of memories [58,59]. We wondered if silencing these DANs will alter the accelerated forgetting in flies expressing *Aβ*_*42*_^*Arctic*^ in the MB. To test this, we genetically silenced MP1, MV1, and V1 DANs by expressing a temperature-sensitive, dominant-negative form of *Dynamin* (*shibire*^*ts*^) using the c150 Gal4 driver [60], while expressing *Aβ*_*42*_^*Arctic*^ using the MB-specific MB247-LexA driver, thus combining the Gal4 and LexA binary expression systems. The expression of shibire^ts^ is further restricted by MBGal80^ts^, a temperature-sensitive repressor of Gal4 [61]. Thus, shibire^ts^ should not affect the MB during training. To inactivate the MB innervating DANs, we transferred flies to the restrictive temperature of shibire^ts^ (30°C, high temperature (HT)) immediately after training for 2 h followed by 4 h at room temperature (RT) (21 to 22°C, RT), while a control group was kept at RT posttraining (6 h at RT). All tested groups showed a similar memory performance when tested immediately after training (Fig 3A-3C). Similarly, when we compared 6-h memory of *c150-Gal4; MB-Gal80* flies that were kept either at RT or HT, they showed similar performance indices (Fig 3B and 3C). As expected, *MB247-LexA*, *LexAop- Aβ*_*42*_^*Arctic*^ flies showed accelerated forgetting, when compared to control flies, and the 6-h memory performance did not differ between the 2 temperature regimes (Fig 3B). However, animals that express *Aβ*_*42*_^*Arctic*^ in the MB and *shibire*^*ts*^ in PPL1 DANs showed a significantly higher memory when exposed to a 2-h HT pulse compared to flies kept continuously at RT (Fig 3C). Thus, genetically silencing the MP1, MV1, and V1 DANs reduce *Aβ*_*42*_^*Arctic*^-induced forgetting. However, the suppressed forgetting might act independently of the *Aβ*_*42*_^*Arctic*^-induced changes. We hypothesize that the changed neuronal activity of MB neurons expressing *Aβ*_*42*_^*Arctic*^ might influence the activity of the forgetting PPL1 DANs.

The forgetting DANs activate the dop1R2 receptor signaling and the downstream RAC pathway [54,55,57,62,63]. We, therefore, tested if impairing RAC signaling affects the accelerated forgetting phenotype, by expressing both *Aβ*_*42*_^*Arctic*^ and a dominant-negative allele of RAC (dRac:DN) together in the MB and test the 2-h aversive memory. We observed that flies expressing both *Aβ*_*42*_^*Arctic*^ and dRac:DN have significantly better performance scores 2 h after conditioning than *OK107> Aβ*_*42*_^*Arctic*^ flies and have normal memory compared to parental control lines (Fig 3D).

### Induced sleep overcomes Aβ-mediated forgetting

**Fig 4:**
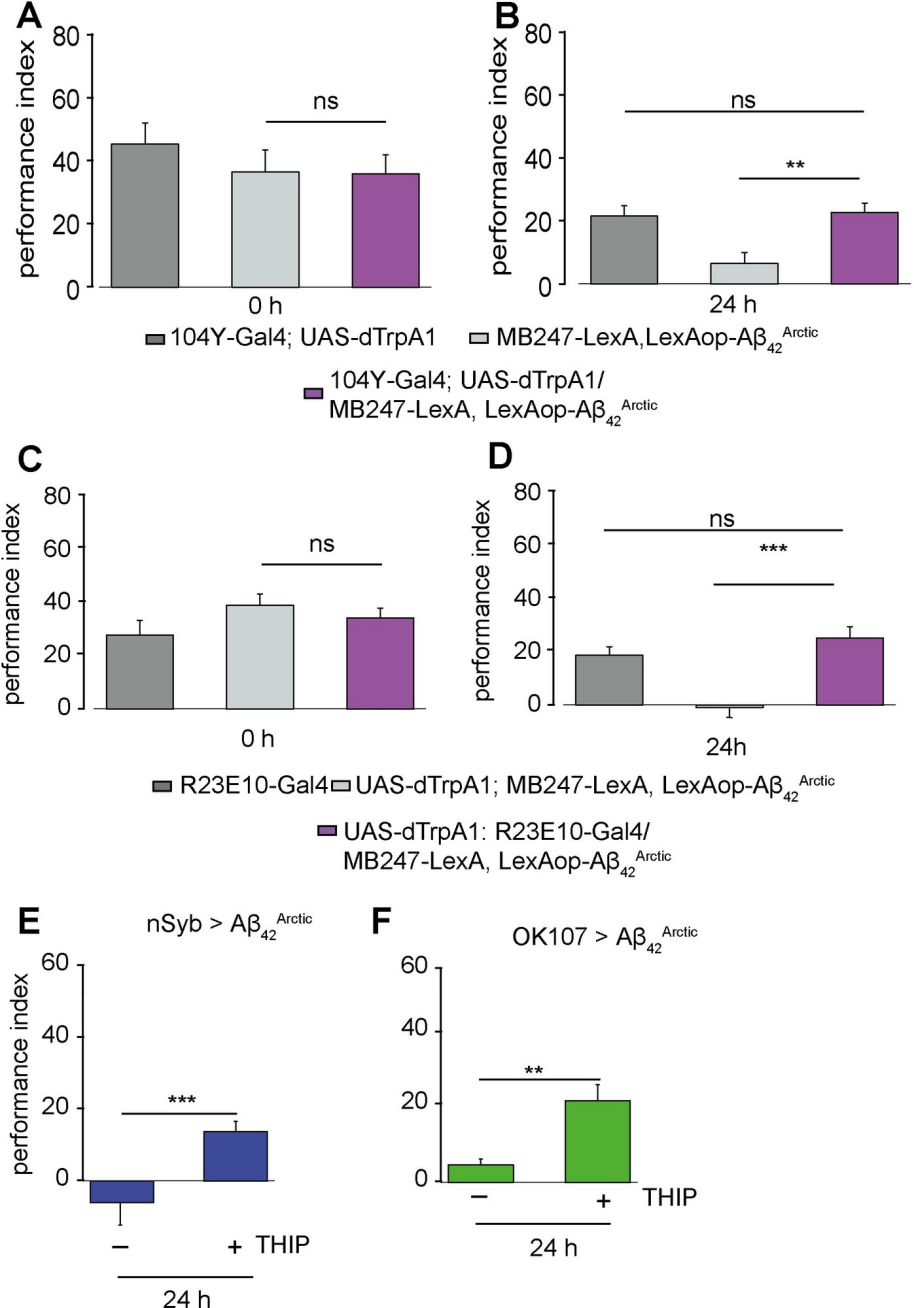
Artificial induction of sleep restores memory impairment in *Aβ_42_^Arctic^* expressing flies. (B, D) Flies were transferred to 29°C after appetitive conditioning to induce sleep by activating dorsal fan-shaped body neurons with *dTrpA1*. (A, B) *dTrpA1* was expressed with *104y-Gal4*. (A) Performance indices of the 3 tested genotypes were not significantly different from each other immediately after conditioning (n ≥ 7). (B) Inducing sleep for 23 h after conditioning could restore 24-h memory in flies, which express *Aβ*_*42*_^*Arctic*^ in the MB (n ≥ 14). (C, D) Sleep was induced by using *R23E10-Gal4*. (C) Directly after conditioning, no significant difference between the tested genotypes was observed (n ≥ 12). (D) 24 h after training, inducing sleep was able to rescue the memory defect of flies expressing *Aβ*_*42*_^*Arctic*^ in the MB (n ≥ 11). (E, F) The drug Gabadoxol (THIP) was used to induce sleep in AD flies. Feeding THIP enhanced LTM tested 24 h after aversive spaced training in flies expressing *Aβ*_*42*_^*Arctic*^ in the brain (E; n ≥ 8) or specifically in the MB (F; n ≥ 8). See S5 Fig for the effect of THIP on sleep and S1 Table for the data. All other details are similar to Fig 1. AD, Alzheimer disease; LTM, long-term memory; MB, mushroom body.

Previous studies in *Drosophila* suggest that sleep can help to consolidate memories by acting on the forgetting circuit and thereby slowing down the forgetting rate [64]. In addition, inducing sleep in fly AD models was shown to be beneficial [65,66]. Moreover, in mammals, sleep can largely restore hyperexcitability caused by the overexpression of *Aβ* peptides [50] and restricts neuronal excitability to a tight physiological range [67]. We thus tested if sleep may be able to modulate the accelerated forgetting caused by *Aβ* peptides. To genetically induce sleep, we expressed *UAS-TrpA1*, a temperature-sensitive cation channel able to activate neurons [68], under the control of the *104y-Gal4* driver line, which is expressed in sleep-promoting dorsal fan-shaped body neurons [69,70]. Experimental and control groups were shifted to 29°C to activate TrpA1, directly after training until 1 h before testing. As expected, overexpression of *Aβ*_*42*_^*Arctic*^ in *MB247-LexA>LexAop- Aβ*_*42*_^*Arctic*^ resulted in an accelerated memory decay after 24 h but not immediately after olfactory conditioning (Fig 4A and 4B). Interestingly, when we induced sleep in AD flies, the LTM decay was restored, suggesting that sleep can improve the accelerated forgetting (Fig 4B). To further validate this finding, we used another Gal4 driver, R23E10-Gal4 [71,72], to activate the sleep neurons by expressing *UAS-TrpA1*. With this driver, we also saw a similar improvement in memory decay (Fig 4C and 4D).

As further validation, we fed flies different concentrations of Gaboxadol (THIP), which induces sleep as observed here and reported earlier (S6A and S6C Fig) [64–66]. It also induced sleep effectively in our *Ok107>Aβ*_*42*_^*Arctic*^ line (S6B Fig). We chose the concentration of 0.01 mg/ml, which was the lowest sleep-inducing concentration and is known to facilitate memory consolidation in different fly models from classical learning mutants to different AD models [64]. Flies were given THIP 1 day prior to aversive spaced training and until the memory performance was evaluated 24 h later. We found that *nSyb> Aβ*_*42*_^*Arctic*^ or *OK107> Aβ*_*42*_^*Arctic*^ flies that were THIP fed showed a significantly higher memory performance after 24 h than flies that did not receive THIP (Fig 4E and 4F). Nevertheless, THIP might affect the locomotion activity of the fly rather than sleep. Previous reports show that THIP-induced sleep fulfills all criteria to be defined as sleep and does not interfere with locomotion [66]. Thus, we monitored the arousal ability of flies after sleep disruption. Therefore, we gave a light pulse at 18 ZT. In both control and THIP-fed groups, more flies were awake during the night (S6C Fig). During the daytime, the 2 groups are not significantly different (S6C Fig).

Moreover, it is described that pan-neuronal *Aβ* peptides cause sleep loss at a young age [50,73]. We observed that flies expressing *Aβ* peptides only in the MB show no obvious sleep defect at the age using for experiments (S6D Fig). However, we cannot exclude that there are subtle changes we do not see with the Trikinetics DAM system. Thus, our result suggests that sleep plays an important role in preventing memory decay caused by *Aβ* peptides.

## Discussion

Here, we show that restricted expression of *Aβ* peptides to the MB of the fly in contrast to pan-neuronal expression does not interfere with memory acquisition. However, memory 2 h after conditioning or later was impaired. This implicates that the flies have a faster memory decay—accelerated forgetting. This phenotype can be at least partially rescued by (1) reducing neuronal activity by genetic or pharmacological means; (2) decreasing the Rac-mediated forgetting pathway; and (3) promoting sleep. Therefore, the results highlight the importance of neuronal homeostasis and sleep in the progression rate of AD.

### Restricted Aβ expression as an early-stage AD model

The early stages of AD are of particular interest as the initiation and early progression of the disease are not well understood. It is believed that the disease is initiated years before symptoms are visible. Therefore, early stages are promising targets for therapeutic interventions to decelerate disease processing [2,3,74].

We propose that restricted *Aβ* expression to the MBs display similarities with an early stage of AD. In contrast to pan-neuronal expression, *Ok107> Aβ*_*42*_^*Arctic*^ or restricted *Aβ*_*42*_^*Arctic*^ expression only in adult flies does not produce symptoms associated with late AD stages like locomotion defects or severe memory impairment. Nevertheless, we cannot rule out that other behaviors are affected in these flies. Likewise, AD patients start losing STM and recent memories early in the disease while keeping stable consolidated LTMs until later stages [2,75–77]. A recent study also showed that familial AD patients show accelerated forgetting [78,79]. Moreover, *Aβ* accumulation seems to begin in the hippocampus, which is like the MB involved in learning and memory and thus shares functional analogies. During disease progression, *Aβ* plaques and NFTs spread in a stereotypic pattern across the brain causing more severe phenotypes [51,80–82]. In our models, it seems that the symptoms increase with age, as longer aged *Ok107> Aβ*_*42*_^*Arctic*^ flies start to show learning impairment. But more observations and experiments are required. All in all, restricted *Aβ* expression can serve as an early-stage AD model.

### AD and neuronal excitability

Mammalian models and brain imaging studies in AD patients indicate that the progression of the disease interferes with neuronal excitability and homeostasis. Patients with late-stage AD show hypoexcitability, whereas patients with MCI, a pre-stage of AD or early AD, show hyperexcitability [6,44,46,47,51,83,84]. A recent study in *Drosophila* using ex vivo live imaging [45] suggests that *Aβ* peptides are causing higher neuronal excitability. The anticonvulsant drug LEV is well known to reduce neuronal excitability. The drug is able to rescue AD phenotypes in rodent models and shows promising results in humans [46–49]. In our model, LEV is also able to rescue the accelerated forgetting phenotype. These results indicate that LEV has a similar effect in the fly and mammals. However, LEV might have more widespread effects on the whole body or indirect effects [85] In addition, the fly metabolic system varies greatly from the mammalian one, so the drug might be differently metabolized. Reassuring, the accelerated forgetting phenotype is also rescued when blocking the neurons expressing *Aβ*_*42*_^*Arctic*^ with *dORK*. However, the rescue is not completely back to wild-type levels. As we manipulate multiple neurons within the MB, we might silence neurons that are required to be active at this time point. However, the *dORK* line we are using is reported to be low expressing and is not completely silencing all the neurons [86–88] so the silencing might not be efficient enough for a complete rescue. Moreover, we cannot exclude that upon the long-term expression of these constructs, compensatory mechanisms or developmental defects occur. Nevertheless, both a pharmacological approach with LEV as well as genetically silencing the *Aβ*_*42*_^*Arctic*^-expressing neurons slows down the memory decay, thereby restoring memory performance. This indicates that *Aβ*_*42*_^*Arctic*^ has similar effects in *Drosophila* as in mammals, making the fly a great model to study AD. Therefore, it will be useful to see how specific neurons change their activity upon *Aβ*_*42*_^*Arctic*^ expression and to manipulate neurons more precisely.

An open question is how *Aβ*_*42*_ peptides could interfere with neuronal excitability. A recent study in a mouse AD model showed that *Aβ*_*42*_ peptides up-regulate Na_v_-channels leading to neuronal hyperexcitability [89]. However, other studies suggest that alteration in the CREB signaling pathway by *Aβ* causes defects in synaptic plasticity and memory formation [90,91]. A further possibility of AD to modify neuronal excitability and plasticity is by down-regulation of repressor element 1-silencing transcription factor (REST), a transcription regulator required for reducing hyperexcitability and restoring neuronal homeostasis [92–94]. All in all, *Aβ*_*42*_ peptides have multiple putative targets in synapses to modify neuronal excitability.

### AD and forgetting circuitry

Forgetting is an active process. In the fly, ongoing activity of a defined set of DANs from the PPL1 cluster after training activates Rac-cofilin signaling in downstream neurons causing forgetting [54–57,63]. *Ok107>Aβ*_*42*_^*Arctic*^ flies have accelerated forgetting, and silencing these forgetting DANs improves the phenotype. Giving that these DANs are both upstream and downstream of the MB [54,58,95], the putative hyperexcitability of *Aβ*_*42*_^*Arctic*^-expressing neurons could enhance the activity of those DANs. This could lead to an overactivation of forgetting signaling. A recent study showed that Rac is elevated in AD patients, mouse models, and fly models [79]. Furthermore, they showed that inhibiting Rac rescues the AD phenotypes. In this study, we show that expressing the dominant-negative Rac allele can rescue the accelerated forgetting phenotype of restricted *Aβ*_*42*_^*Arctic*^ expression. Thus, the involved mechanism seems to be quite similar in flies and mammals.

However, Rac or other components of the pathway could also be increased due to intrinsic changes in MB neurons caused by *Aβ*_*42*_^*Arctic*^. Careful monitoring of the activity of DANs and KCs as well as analyzing other pathway members—for example, the dop1R2/Damb dopamine receptor or cofilin—could shed light on this. Moreover, the Rac-signaling pathway is not the only regulator of learning and forgetting. Whereas the Rac-pathway is implied in regulating ASM cdc42 is involved in ARM. Furthermore, learning activates the Raf/MAPK pathway to suppress forgetting [53,96,97]. Hence, there might be multiple possibilities of how *Aβ*_*42*_^*Arctic*^ can enhance forgetting. Nevertheless, we cannot exclude that *Aβ*_*42*_^*Arctic*^ in the MB interferes with memory consolidation and engram formation.

### AD and sleep

Sleep disruption is a commonly observed phenotype in AD patients. Different studies showed that promoting sleep and improve sleep quality can improve disease [2,3,98–103]. In *Drosophila*, sleep was able to rescue the memory defects of AD flies [65,66]. In this study, sleep is also able to rescue the accelerated forgetting phenotype.

Sleep is an important regulator of brain function. It is implemented in memory consolidation and regulation of neuronal homeostasis. Neuronal homeostasis refers to changes in neuronal or synaptic properties to maintain a level of activity across the whole nervous system [104,105]. Further, sleep has been shown to be beneficial for *Aβ* clearance in mammalian AD models [100–102,106]. Therefore, AD has a bidirectional relationship with sleep. Although the MB functions in sleep regulation [107,108], young *Ok107>Aβ*_*42*_^*Arctic*^ flies seem to exhibit normal sleep. We suggest that the dorsal fan-shaped body, another brain region for sleep regulation, especially sleep homeostasis [71,108,109] is compensating for the alterations in the MB sleep network. Another explanation would be that the amyloid load in the MB is not severe enough to disrupt the MB sleep circuits. However, more studies are required to understand the function of the sleep circuitry in our model and its relationship to *Aβ* peptides.

Interestingly, a study in *Drosophila* showed that sleep suppresses the activity of the DANs involved in forgetting and that THIP-induced sleep suppresses this forgetting [64]. Therefore, inducing sleep in our model might reduce the excitability of the DANs and MB circuit to rescue the observed AD phenotype.

In our experiments, the anticonvulsant drug LEV is not able to able to rescue the memory defect of *nSyb>Aβ*_*42*_^*Arctic*^ flies, whereas enhancing sleep with THIP is. THIP-induced sleep was shown to fulfill the classical criteria for sleep. Previous work in *Drosophila* has shown that inducing sleep rescues AD in both a Presenilin-based model, a tau model, and a coexpression model of APP and BACE [65,66,110,111]. We hypothesize that the brain networks in the nSyb> *Aβ*_*42*_^*Arctic*^ flies might be too disrupted to be simply rescued by lowering neuronal activity, whereas sleep could modify the network with more precise regulations. However, maybe longer feeding of LEV or an optimized dose could have a positive effect. Although THIP is used as a sleeping drug, we cannot exclude that it has other effects that contribute to the observed rescue.

Interestingly, Tau, another protein involved in AD and also other types of dementia, interferes with sleep, neuronal excitability, and homeostasis in mammalian models [100,101,112,113]. Therefore, untangling the relationship between AD, sleep, and neuronal homeostasis might be a path to finding a therapeutic approach.

### Beyond AD

Due to its easy handling, short generation time, and available genetic tools, *Drosophila* is a widely used model organism. The fly’s neuronal system is simple enough to manipulate it while allowing different kinds of behaviors. Because most human genes linked to diseases as well as basic molecular processes of the brain are conserved, the fly is used as a model for neurodegenerative diseases. Most studies of AD in flies use the pan-neuronal expression of *Aβ*_*42*_. However, our model used here has restricted *Aβ*_*42*_^*Arctic*^ expression in neurons relevant for learning. Therefore, we can monitor the effect of *Aβ*_*42*_^*Arctic*^ on specific neuropil and its features. Moreover, by using tools like genes switch or gal80ts, the expression could be even more controlled to study *Aβ*_*42*_^*Arctic*^ accumulation. Additionally, by coexpressing other RNAi lines or genetic tools, modifying genes of the accelerated forgetting could be discovered. This could potentially help to find interaction partners of *Aβ*_*42*_^*Arctic*^ in the neurons. Lastly, the model could be used to test other drugs. Nevertheless, there are also disadvantages. Firstly, our model is based on overexpression, so disease initiation is different than in humans. Further, not all features and symptoms of AD are present in the fly. Moreover, the human brain and the cognitive/behavioral functions are more complicated than in *Drosophila*. Another factor to consider is that the flies have a completely different digestive system and circulation, so drugs most likely have different kinetics, different effects/targets, and are differently metabolized.

The results shown here can also be used as a starting point to understand neuronal excitability better. Although there is ample evidence that local and global changes in excitability play a role in all steps of memory processing, there are still gaps in our knowledge. In this study, increasing the intrinsic excitability of the MB neurons seems to disrupt the consolidation of LTM and/or initiating the forgetting pathway. Silencing downstream neurons or manipulating global excitability levels via sleep seems to improve the observed phenotype.

## Material and methods

### Lead contact

Further information and requests for resources and reagents should be directed to and will be fulfilled by the Lead Contact, Simon Sprecher (simon.sprecher@unifr.ch).

### Experimental model and subject details

#### Fly husbandry

*Drosophila melanogaster* flies were reared in plastic vials on standard cornmeal food (12 g agar, 40 g sugar, 40 g yeast, 80 g cornmeal per liter) and transferred to fresh food vials every 2 to 3 days. Flies were generally kept at 25°C, 60% to 65% humidity, and exposed to 12-h light and 12-h darkness with light onset at 8 AM. *OK107-Gal4* (106098) was obtained from the Kyoto stock center. *nSyb-Gal4* (51635), *UAS-shibire*^*ts1*^ (44222), *UAS-dTrpA1* (26263), *UAS-dOrk1*.*ΔC2* (6586), *UAS-dOrk1*.*ΔNC* (6587), and *GMR23E10-Gal4* (49032) were received from the Bloomington stock center. *c150-Gal4*; *MB-Gal80* was obtained from Alex Keene (Florida Atlantic University) [114]. *MB-Gal4*^*GS*^ (FlyBase ID: FBtp0015149), *UAS- Aβ*_*42*_^*Arctic*^, *UAS-Aβ*^*40*^, *104Y-Gal4* (FlyBase ID: FBti0072312), and *MB247-LexA LexAop-Aβ*_*42*_^*Arctic*^ were gifted to us by Mark Wu and Andrew Lin (Johns Hopkins University, University of Sheffield). *Elav-Gal4*^*GS*^ was obtained from Frank Hirth (King’s College London). Iso31 [115] was used as a wild-type strain. The experimental lines were generated by crossing the UAS and Gal4 constructs together. At the same time, the parental lines were crossed with Iso31 to have 1 copy of Gal4 or UAS like in the experimental strain. Moreover, Iso31 was always tested in parallel as a control.

#### Learning apparatus

For behavior experiments, we used a memory apparatus that is based on Tully and Quinn’s design and modified it to allow conducting 4 memory experiments in parallel (CON-Elektronik, Greussenheim, Germany). Experiments were performed at 23 to 25°C and 65% to 75% relative humidity. The training was performed in dim red light, and memory tests were done in complete darkness. The 2 odors used were 3-Oct (Sigma-Aldrich Cat# 218405-250G; CAS Number: 589-98-0) and MCH (Sigma-Aldrich Cat# 66360-250G; CAS Number 589-91-3) diluted in paraffin oil (Sigma-Aldrich Cat# 18512–2.5L; CAS Number 8012-95-1) 1:100, respectively. A volume of 260 μl of the diluted odors were presented in a plastic cup of 14 mm in diameter. A vacuum membrane pump ensured odor delivery at a flow rate of 7 l/min.

#### Aversive olfactory conditioning

For aversive conditioning, groups of 50 to 100 flies with mixed sex were loaded in tubes lined with an electrifiable copper grid. Position in the machine and the sequence in which the genotypes were tested were randomized. Experiments in which more than half of the flies died, the flies did not move or there were technical problems with the machine, as well as human errors were excluded. The training was conducted in the morning. After an accommodation period of 90 s, the first odor was presented for 60 s. In parallel, 12 pulses of 100 V for 1.5 s were delivered with an interval of 3.5 s. After 30 s of flushing with fresh air, the second odor was presented for 60 s. For the subsequent group of flies, the order of the 2 odors was reversed. For measuring 0-h performance, flies were tested about 3 min after the end of the conditioning. To determine 2-h, 4-h, and 6-h memory performance, flies were transferred to food vials after conditioning and kept at 25°C until the test.

#### Appetitive olfactory conditioning

Before appetitive conditioning, groups of 50 to 100 flies with mixed sex were starved for 19 to 21 h in plastic vials containing damp cotton at the bottom. Experiments in which more than half of the flies died, the flies did not move or there were technical problems with the machine, as well as human errors were excluded. Position in the machine and the sequence in which the genotypes were tested were randomized. The training was conducted in the morning. The conditioning protocol consists of a 90-s accommodation period, 120 s of the first odor, 60 s of fresh air followed by 120 s of the second odor. During the first odor, flies are in a conditioning tube lined with filter paper that was soaked in water the day before the experiment and left to dry overnight. For the second odor, flies are transferred to a conditioning tube lined with a filter paper that was soaked with a 1.5 M sucrose (Sigma-Aldrich, Cat# 84100-1KG; CAS Number 57-50-1) solution on the day before and left to dry at RT. After conditioning, flies were either directly tested for STM or put back in starvation vials until the memory test 2 h or 6 h later. For 24-h memory, flies were fed for 3 h after training before starving them again. One experiment consisted of 2 reciprocal conditionings, in which the odor paired with sucrose was reversed.

#### Memory tests

The memory test is identical for aversive and appetitive conditioning. Flies were loaded into a sliding compartment and transferred to a two-arm choice point. Animals were allowed to choose between 3-Oct and MCH. After 120 s, flies trapped in both arms were collected separately and counted. Based on these numbers, a preference index was calculated as follows:

PREF = ((N_arm1_ − N_arm2_) 100) / N_total_ the 2 preference indices were calculated from the 2 reciprocal experiments. The average of these 2 PREFs gives a memory performance index (PI). PI = (PREF_1_ + PREF_2_) / 2.

#### Cold shock experiments

After conditioning, flies were transferred into precooled plastic vials that were then placed in ice water for 2 min. Afterward, flies were moved to food vials and kept at 25°C until the memory test.

#### Sensory accuracy tests

Flies were tested for their ability to sense the 2 used odors 3-Oct and MCH as well as electric shock and sugar. Therefore, the flies were loaded into a sliding compartment and brought to a two-arm choice point. The flies were allowed to freely choose between an arm containing the stimulus and a neutral arm. All experiments were carried out in the dark. Afterward, the flies in each arm were counted, and a preference index was calculated.

For testing the odor response, the flies could choose between one of the odors in the same concentration as used for the behavior experiment and the same amount of paraffin oil for 120 s.

Preference index PI = ((N_air_ − N_odor_)100) / N_total_.

For shock response, the flies could freely choose between a cooper grid–lined tube getting pulses of 100 V for 60 s or a cooper grid–lined tube getting no electric shock. Preference index PI = ((N_No shock_ − N_shock_)100) / N_total_.

For testing sugar sensitivity, a group of flies was starved for 1 to 21 h in a tube with damp cotton on the bottom. They could choose for 120 s between a tube lined with filter paper that was soaked in 1.5 M sucrose solution the day before or a tube lined with filter paper that was soaked in distilled water the day before. Preference index PI = ((N_sucrose_ − N_water_)100) / N_total_.

#### GeneSwitch Gal4 system

Mifepristone (Ru486) (Sigma-Aldrich, Cat# M8046-100MG; CAS Number 84371-65-3) was dissolved in 100% EtOH and added to a total concentration of 250 μM to the fly food. Flies were fed RU486 food for 7 days before conditioning. The control group received food, in which only the vehicle (EtOH) was added for 7 days.

#### Modulation of neuronal activity

Temporal neuronal silencing was carried out by the expression of *shibire*^ts^, which blocks neurotransmission at the restrictive temperature of 30°C, while at the permissive temperature of 21 to 22°C, neurons remain unaffected [60]. For neuronal silencing experiments, flies were moved immediately after training to 30°C for 2 h, followed by 4 h at 21 to 22°C. For control experiments, flies were kept at 21 to 22°C for 6 h.

*UAS-dTrpA1* was used to activate specific neurons. Temperature above 25°C induces stimulation of neurons ectopically expressing *dTrpA1* [68]. For experiments with *UAS-dTrpA1*, flies were raised at 23°C. Flies were transferred to HT (29°C) after training and were returned to 23°C 1 h before testing. For 24-h memory, flies were exposed to 29°C for 23 h and then transferred to 23°C 1 h before the test.

*UAS-dOrk1*.*ΔC* was used to silence neurons, whereas *UAS-dOrk1*.*ΔNC* was used as a control.

#### Drug administration

LEV (Sigma-Aldrich, Cat# L8668-50MG; CAS Number: 102767-28-2) was added to the fly food at a concentration of 5 mg/kg, as described by Tabuchi and colleagues [50]. Further concentrations tested were (1, 2, 3, 4 mg/kg). *nSyb* > *Aβ*_*42*_^*Arctic*^ and the corresponding parental controls were collected 1 day after hatching and maintained on food containing LEV for 3 to 4 days. For experiments with the driver *OK107-Gal4*, 1-day-old flies were transferred into food vials with LEV and maintained on this food for 7 days. At the end of the LEV-feeding period, olfactory conditioning experiments were performed. After the training, flies were put back on food with LEV until the test.

Gaboxadol, also known as 4,5,6,7-tetrahydroisoxazolo (5,4-c) pyridin-3-ol (THIP) (Sigma-Aldrich, Cat# T101-100MG; CAS Number 85118-33-8), was added to the food to reach concentrations of 0.01, 0.06, or 0.33 mg/ml. For conditioning experiments, the concentration of 0.01 mg/ml was used, and flies were placed on THIP food 1 day prior to conditioning and continued to be on THIP food until the test. For sleep recording experiments, the locomotor tubes contained food with different concentrations of THIP.

#### Sleep experiments

Four-day-old male flies were loaded in 5 × 65 mm plastic tubes containing food on one side. Flies were recorded using the Drosophila Activity Monitoring System (www.trikinetics.com) in an incubator at 25°C with a 12-h light–12-h darkness cycle. Sleep was defined as 5 min of continuous rest, and the sleep data were analyzed using pySolo software [116].

#### Whole-mount Aβ_42_ immunostaining

For dissections and whole-mount Aβ42 immunostaining, the STAR protocol by Sekiya and Iijima was followed with minor modifications [32]. In brief, adult flies were anesthetized on ice, and the brains were dissected in cold 1X PBS (BioFroxx 1346LT050). After a maximum of 30 min, they were fixed for 1 h at RT in 4% paraformaldehyde in PBS and then washed with 1X PBS containing 0.5% Triton X-100 (Carl Roth 3051.3) (0.5% PBST). The permeabilized brains were treated with 10% formic acid (Sigma-Aldrich F0507-100ML) for 1 h, and then blocked (10% NGS, 1% Triton X-100, 1X PBS) before adding the primary mouse anti-Aβ 6E10 (BioLegend Cat # 803001), 1:200) and incubating overnight at 4°C in the dark. The secondary antibody, goat Alexa Fluor 488-conjugated anti-mouse IgG (1:500) (Molecular Probes A11029), was also added overnight. The brains were mounted using self-made mounting media (90% Glycerol (Fischer Scientific Catalog No. BP229-1), 0.5% N-propyl gallate (Sigma P3130), 20 mM Tris (Fischer Scientific, Catalog No. BP152-5), pH 8.0) (Adapted from NIC Harvard Medical School).

The brains were imaged using a confocal microscope (Leica STELLARIS 8 FALCON) at 63X magnification with the HC PL APO CS2 63x/1.30 GLYC objective, and all were taken using the same parameters. Images were analyzed using Fiji [117], where a Z-project of 75 slices at maximum intensity was taken for each image. Then, 3 identical ROIs were measured (one for each MB and one for the background). In Microsoft Excel, the background intensity was subtracted from the MB measurements, and then they were averaged (2 measurement per image, 2 to 4 images per condition), and the SEM was calculated. The average intensity for each condition was then plotted in R using ggplot2.

### Statistical analysis

To compare performance indices or sleep between different groups, we used one-way analysis of variance (ANOVA) with post hoc Tukey honestly significant difference (HSD) test calculator for comparing multiple treatments in R with the package multcomp. In the case of 2 groups, we performed a *t* test for comparison. Data are available in S1 Table.

## Supporting information

S1 Fig Sensory accuracy test of *Aβ_42_^Arctic^-*expressing flies. **Related to Fig 1.** (A-C) Sensory tests of flies expressing *Aβ*_*42*_^*Arctic*^ pan-neuronally alongside the parental controls. (A) Odor avoidance test of MCH. (B) Odor avoidance test for 3-Oct. (C) Shock response test. (D) 2-h aversive memory of *nSyb > Aβ*_*42*_^*Arctic*^ flies is significantly different to parental controls. (E-G) Sensory test of flies expressing *Aβ*_*42*_^*Arctic*^ in the MB alongside the parental controls. (E) Odor avoidance test of MCH. (F) Odor avoidance test for 3-Oct. (G) Shock response test. (H) Memory assessment of flies expressing *Aβ*^*Arctic*^ in the MB at different time points (0, 2, 4, and 6 h) after conditioning. The values for 0 h and 2 h are the same as in Fig 1C and 1D. (I) Flies were subjected to 2 min of cold shock treatment that erases the labile ASM component. Animals expressing *Aβ*_*42*_^*Arctic*^ in the MB showed impaired 2-h memory after cold shock conditions (n ≥ 12). ASM, anesthesia-sensitive memory; MB, mushroom body; MCH, 4-methyl-cyclohexanol; 3-Oct, 3-Octanol.
(TIF)

S2 Fig Sensory accuracy test of *Aβ_42_^Arctic^-*expressing flies using the GeneSwitch system. **Related to Fig 1.** (A-C) Sensory tests of flies expressing *Aβ*_*42*_^*Arctic*^ restricted to the adult stage with Elav^GS^ alongside the parental controls. Flies were fed with either RU486 or the vehicle. (A) Odor avoidance test of MCH. (B) Odor avoidance test for 3-Oct. (C) Shock response test memory scores of the tested genotypes did not differ from each other. (D) 0-h aversive memory of flies expressing *Aβ*_*42*_^*Arctic*^ in the adult brain using the Elav^GS^ system. Flies were fed with either RU486 or the vehicle. (E) 0-h aversive memory of flies expressing *Aβ*_*42*_^*Arctic*^ in the adult MB using the MB^GS^ system. Flies were fed with either RU486 or the vehicle. (F) Memory performance 2 h after aversive training of 4- to 5-day-old *OK107 > Aβ*_*42*_^*Arctic*^ flies and parental controls with no accelerated forgetting phenotype. (G) 14- to 15-day-old *OK107 > Aβ*_*42*_^*Arctic*^ flies show a learning defect compared to parental controls. All other details are similar to Fig 1. MB, mushroom body; MCH, 4-methyl-cyclohexanol; 3-Oct, 3-Octanol.
(TIF)

S3 Fig Sugar sensing capability of *Aβ_42_^Arctic^-*expressing flies. **Related to Fig 1.** (A) Sugar response of flies expressing *Aβ*_*42*_^*Arctic*^ pan-neuronally alongside parental control lines. (B) Sugar response of flies expressing *Aβ*_*42*_^*Arctic*^ in the MB alongside the parental controls. All other details are similar to Fig 1. MB, mushroom body.
(TIF)

S4 Fig Expression of amyloid β peptides in the DANs. **Related to Fig 1.** (A) Aversive olfactory conditioning experiment was conducted with *Aβ*_*42*_^*Arctic*^-expressing flies in most DANs. TH> *Aβ*_*42*_^*Arctic*^ flies have a learning defect after training (n ≥ 12) (B) Appetitive olfactory conditioning with flies expressing *Aβ*_*42*_^*Arctic*^ in the PAM cluster. PAM > *Aβ*_*42*_^*Arctic*^ flies have a learning defect immediately after training (n ≥ 12). See S1 Table for the data. Bar graphs represent the mean, and error bars represent the standard error of the mean. Asterisks denote significant difference between groups (*p < 0.05, **p < 0.005, ***p < 0.001). DAN, dopaminergic neuron.
(TIF)

S5 Fig Effect of LEV on sleep. **Related to Fig 2.** (A) Effect of LEV on the 2-h aversive memory of *nSyb > Aβ*_*42*_^*Arctic*^ flies. The memory impairment is not rescued by LEV. (B) Amount of day and night time sleep of flies receiving different amounts of LEV. (C) Expression of the nonconducting K^+^-channel dORKΔNC or constitutive conducting dORKΔC in the MB of *Aβ*_*42*_^*Arctic*^ -expressing flies. At 0 h after training, no significant difference is observed (n ≥ 8). (D, E) Memory performance of flies only expressing the dORK constructs 0 h (D) and 2 h (E) after training. (n ≥ 8). All other details are similar to Fig 1. LEV, Levetiracetam; MB, mushroom body.
(TIF)

S6 Fig Gabadoxol (THIP) feeding induces sleep. **Related to Fig 4.** (A) Sleep profile of wild-type Canton-S flies that received different concentrations of THIP or the vehicle. (B) Sleep profile of *OK107-Aβ*_*42*_^*Arctic*^ flies and parental controls on THIP (C) Quantification of daytime and nighttime sleep; daytime and nighttime sleep was significantly increased in flies that received 0.01, 0.06, or 0.33 mg/ml THIP (n ≥ 24), compared to control flies that received no THIP. (D) Arousal response of THIP-fed flies. At ZT18, a light pulse was used to wake the flies up. (E) Sleep profile of 8- to 10-day-old flies expressing *Aβ*^*Arctic*^ in the MB. All other details are similar to Fig 1. MB, mushroom body.
(TIF)

S7 Fig Expression of *Aβ_42_^Arctic^* in the brain. **Related to Figs 1 and 3.** (A) Whole-mount immunostaining of Iso31 and *Ok107>Aβ*_*42*_^*Arctic*^ brains with 6E10 antibody. The top row shows untreated flies, and the bottom row brains of flies fed with 5 mg/kg LEV. (B) Quantification of *Aβ*_*42*_^*Arctic*^-expression. LEV, Levetiracetam.
(TIF)

S1 Table Data and statistical analysis for preparing the figures. Related to all figures.
(XLSX)

## Abbreviations

AD: Alzheimer disease
APP: amyloid beta precursor protein
ARM: anesthesia-resistant memory
ASM: anesthesia-sensitive memory
DAN: dopaminergic neuron
HT: high temperature
LEV: Levetiracetam
LTM: long-term memory
MB: mushroom body
MCH: 4-methyl-cyclohexanol
MCI: mild cognitive impairment
MTM: Middle-term memory
NFT: neurofibrillary tangle
REST: repressor element 1-silencing transcription factor
RT: room temperature
STM: Short-term memory
3-Oct: 3-Octanol
