# Supplementary figures and images for: Dopamine, sleep, and neuronal excitability modulate amyloid-β–mediated forgetting in Drosophila
Source: PLoS Biol. 2021 Oct 6;19(10):e3001412. doi: 10.1371/journal.pbio.3001412 (PMC8523056; doi:10.1371/journal.pbio.3001412)

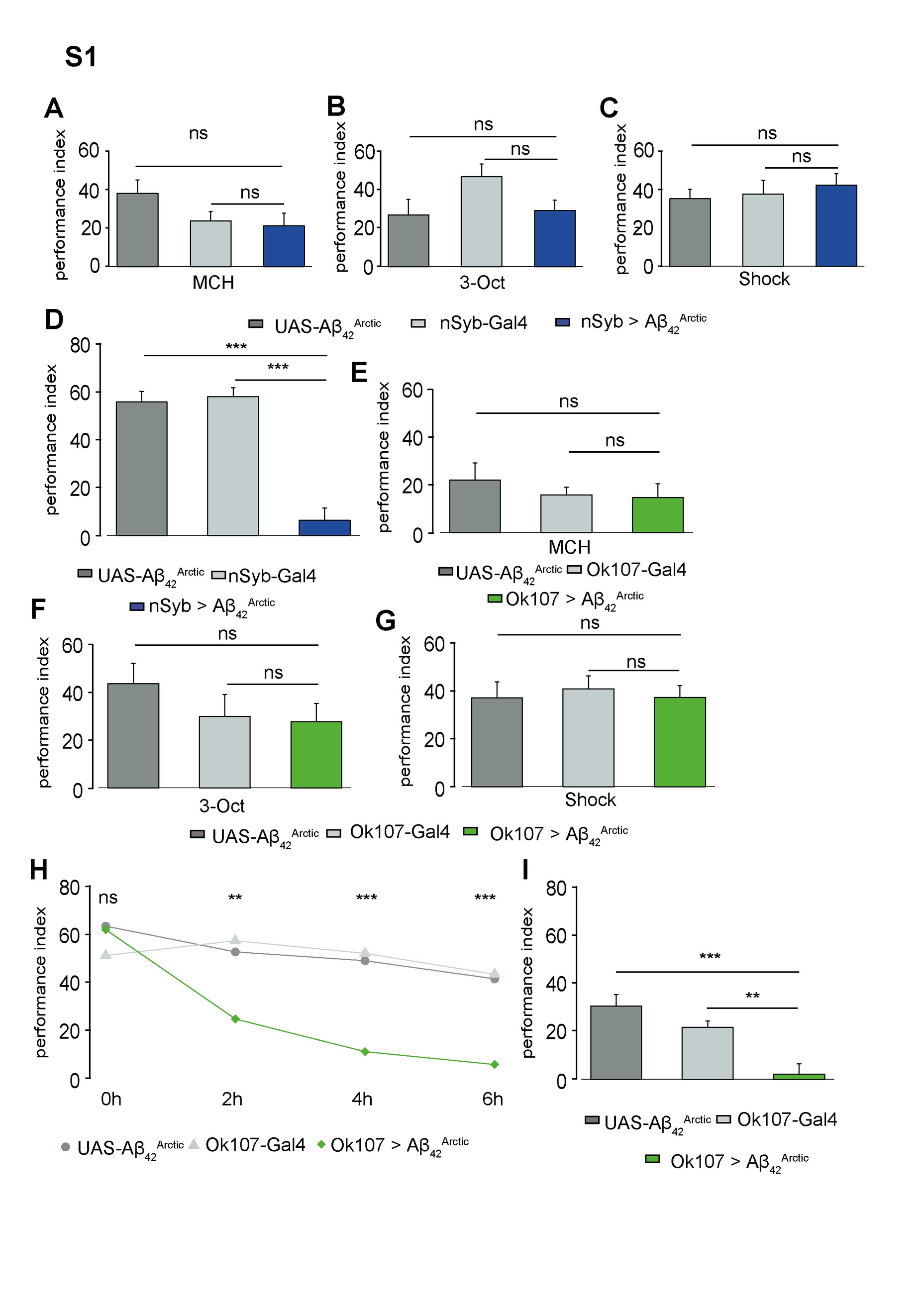

Supplement: S1 Fig — Related to Fig 1. (A-C) Sensory tests of flies expressing Aβ42Arctic pan-neuronally alongside the parental controls. (A) Odor avoidance test of MCH. (B) Odor avoidance test for 3-Oct. (C) Shock response test. (D) 2-h aversive memory of nSyb > Aβ42Arctic flies is significantly different to parental controls. (E-G) Sensory test of flies expressing Aβ42Arctic in the MB alongside the parental controls. (E) Odor avoidance test of MCH. (F) Odor avoidance test for 3-Oct. (G) Shock response test. (H) Memory assessment of flies expressing AβArctic in the MB at different time points (0, 2, 4, and 6 h) after conditioning. The values for 0 h and 2 h are the same as in Fig 1C and 1D. (I) Flies were subjected to 2 min of cold shock treatment that erases the labile ASM component. Animals expressing Aβ42Arctic in the MB showed impaired 2-h memory after cold shock conditions (n ≥ 12). ASM, anesthesia-sensitive memory; MB, mushroom body; MCH, 4-methyl-cyclohexanol; 3-Oct, 3-Octanol. (TIF) [file pbio.3001412.s001.tif]

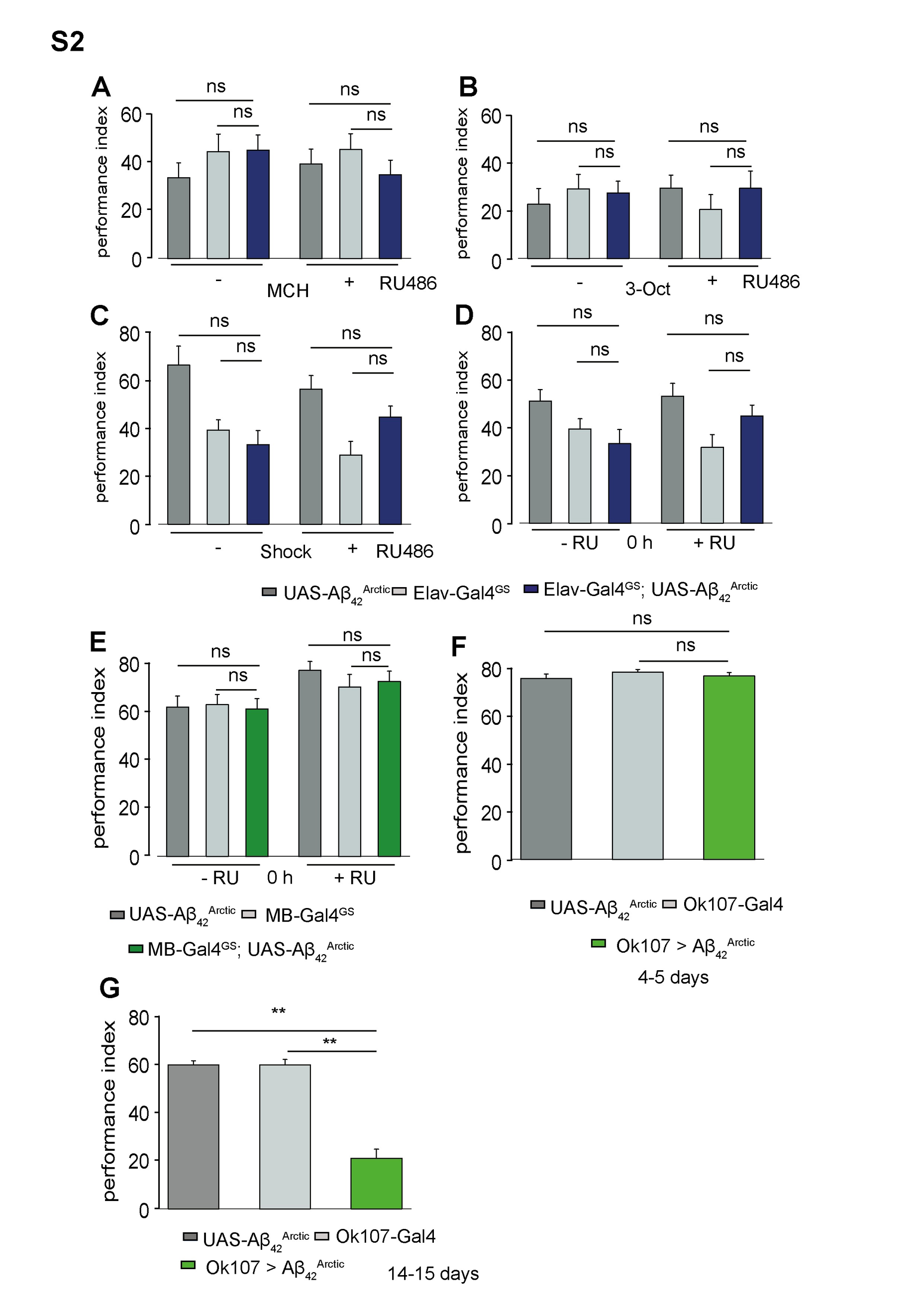

Supplement: S2 Fig — Related to Fig 1. (A-C) Sensory tests of flies expressing Aβ42Arctic restricted to the adult stage with ElavGS alongside the parental controls. Flies were fed with either RU486 or the vehicle. (A) Odor avoidance test of MCH. (B) Odor avoidance test for 3-Oct. (C) Shock response test memory scores of the tested genotypes did not differ from each other. (D) 0-h aversive memory of flies expressing Aβ42Arctic in the adult brain using the ElavGS system. Flies were fed with either RU486 or the vehicle. (E) 0-h aversive memory of flies expressing Aβ42Arctic in the adult MB using the MBGS system. Flies were fed with either RU486 or the vehicle. (F) Memory performance 2 h after aversive training of 4- to 5-day-old OK107 > Aβ42Arctic flies and parental controls with no accelerated forgetting phenotype. (G) 14- to 15-day-old OK107 > Aβ42Arctic flies show a learning defect compared to parental controls. All other details are similar to Fig 1. MB, mushroom body; MCH, 4-methyl-cyclohexanol; 3-Oct, 3-Octanol. (TIF) [file pbio.3001412.s002.tif]

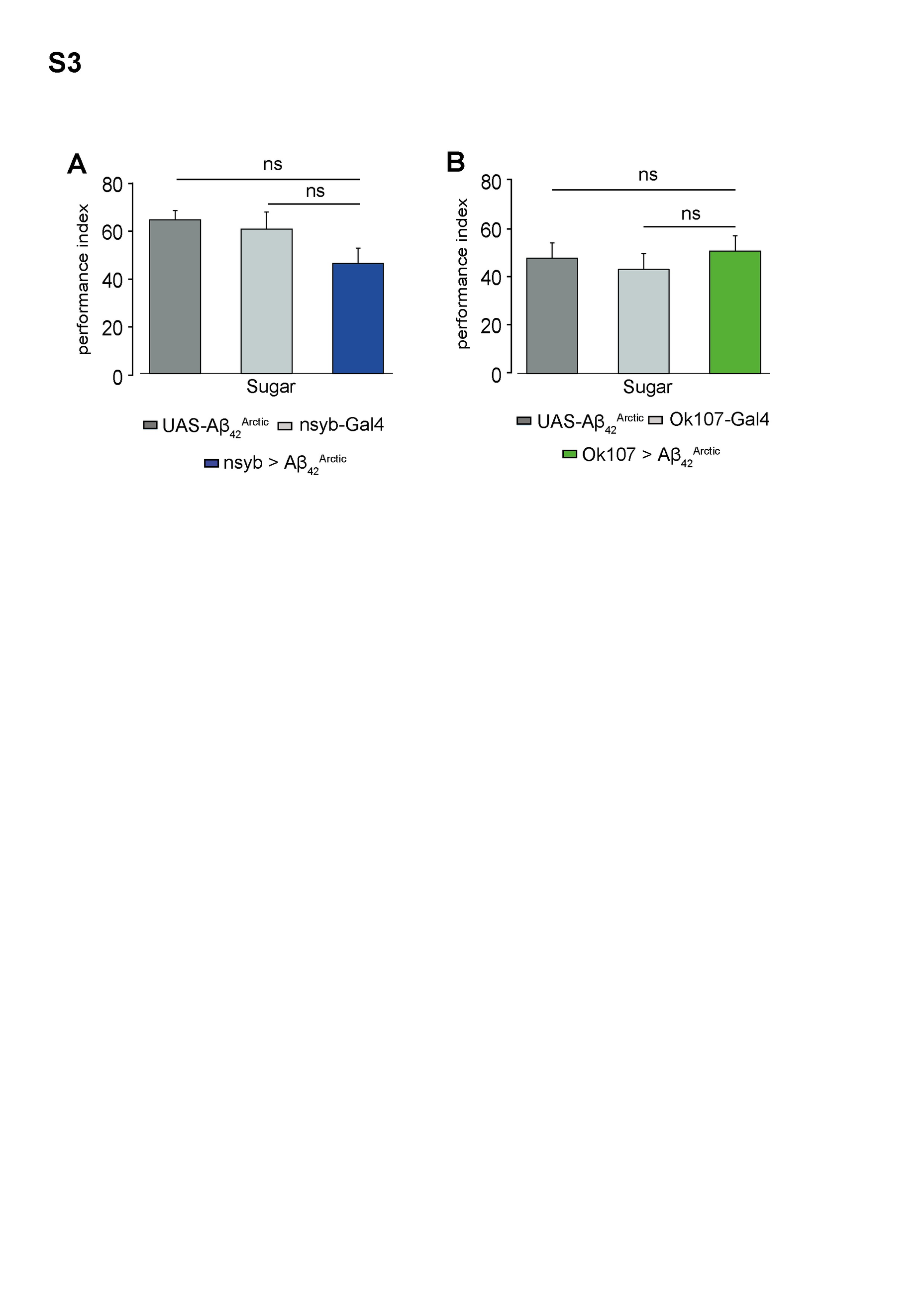

Supplement: S3 Fig — Related to Fig 1. (A) Sugar response of flies expressing Aβ42Arctic pan-neuronally alongside parental control lines. (B) Sugar response of flies expressing Aβ42Arctic in the MB alongside the parental controls. All other details are similar to Fig 1. MB, mushroom body. (TIF) [file pbio.3001412.s003.tif]

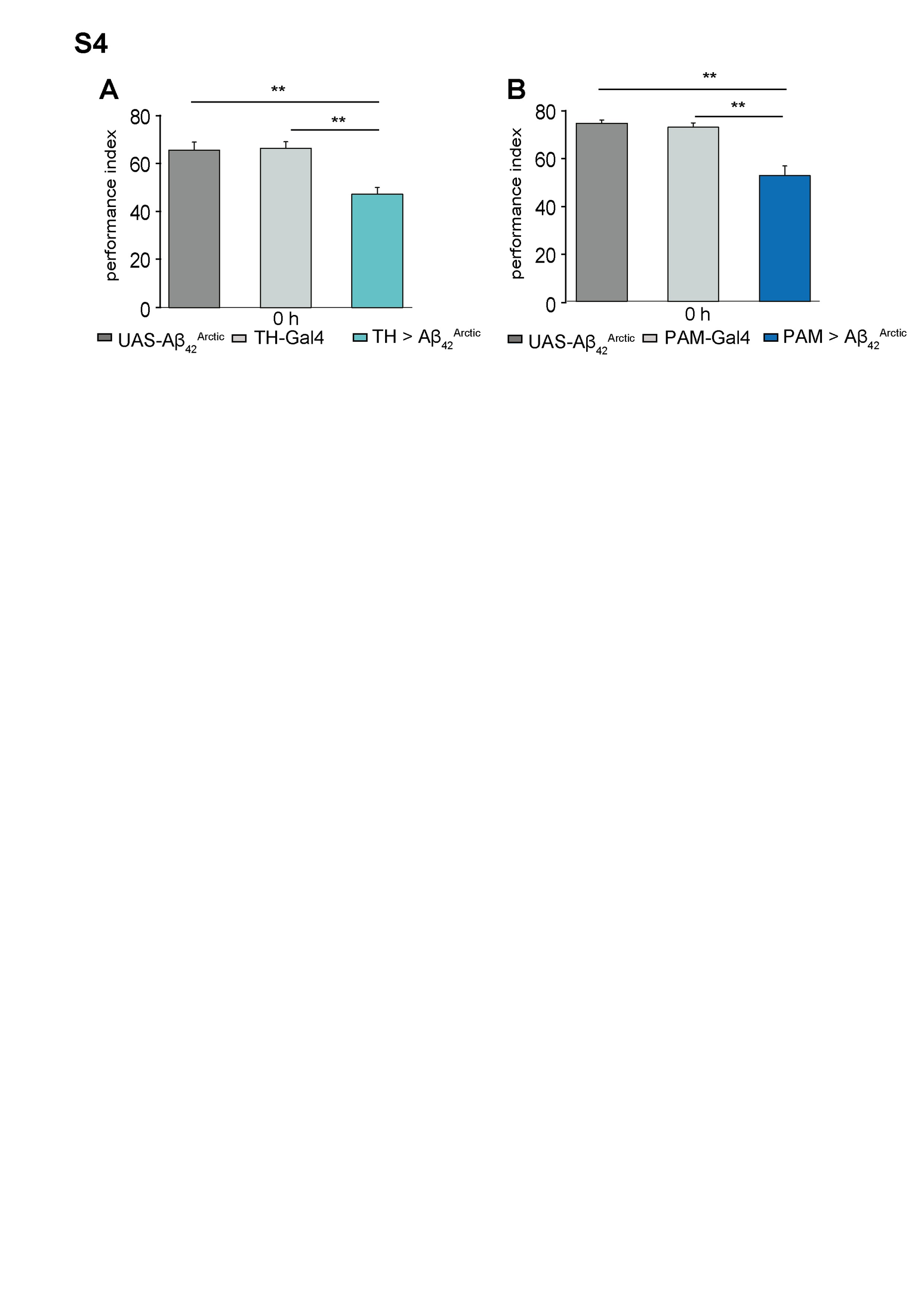

Supplement: S4 Fig — Related to Fig 1. (A) Aversive olfactory conditioning experiment was conducted with Aβ42Arctic-expressing flies in most DANs. TH> Aβ42Arctic flies have a learning defect after training (n ≥ 12) (B) Appetitive olfactory conditioning with flies expressing Aβ42Arctic in the PAM cluster. PAM > Aβ42Arctic flies have a learning defect immediately after training (n ≥ 12). See S1 Table for the data. Bar graphs represent the mean, and error bars represent the standard error of the mean. Asterisks denote significant difference between groups (*p < 0.05, **p < 0.005, ***p < 0.001). DAN, dopaminergic neuron. (TIF) [file pbio.3001412.s004.tif]

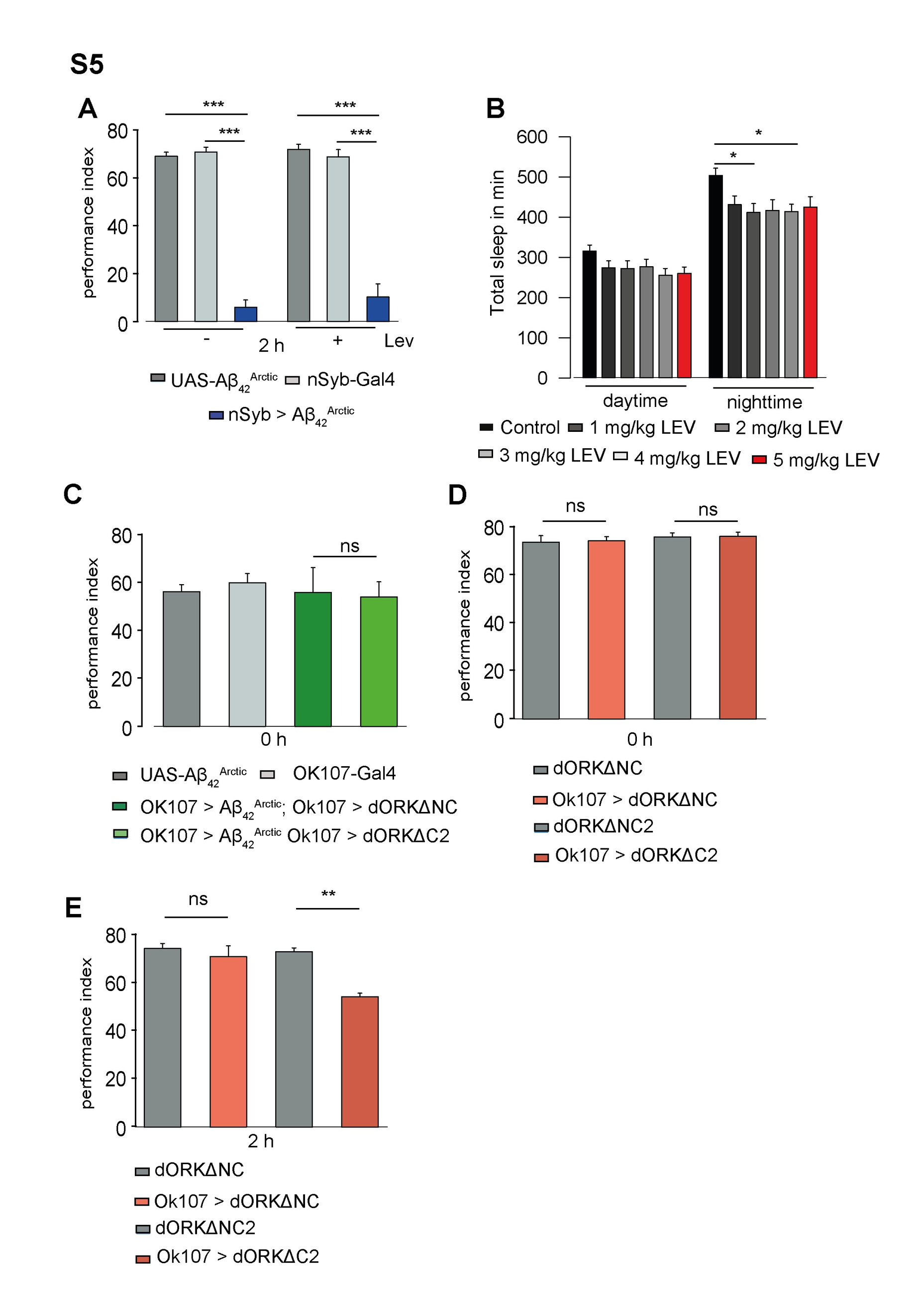

Supplement: S5 Fig — Related to Fig 2. (A) Effect of LEV on the 2-h aversive memory of nSyb > Aβ42Arctic flies. The memory impairment is not rescued by LEV. (B) Amount of day and night time sleep of flies receiving different amounts of LEV. (C) Expression of the nonconducting K+-channel dORKΔNC or constitutive conducting dORKΔC in the MB of Aβ42Arctic -expressing flies. At 0 h after training, no significant difference is observed (n ≥ 8). (D, E) Memory performance of flies only expressing the dORK constructs 0 h (D) and 2 h (E) after training. (n ≥ 8). All other details are similar to Fig 1. LEV, Levetiracetam; MB, mushroom body. (TIF) [file pbio.3001412.s005.tif]

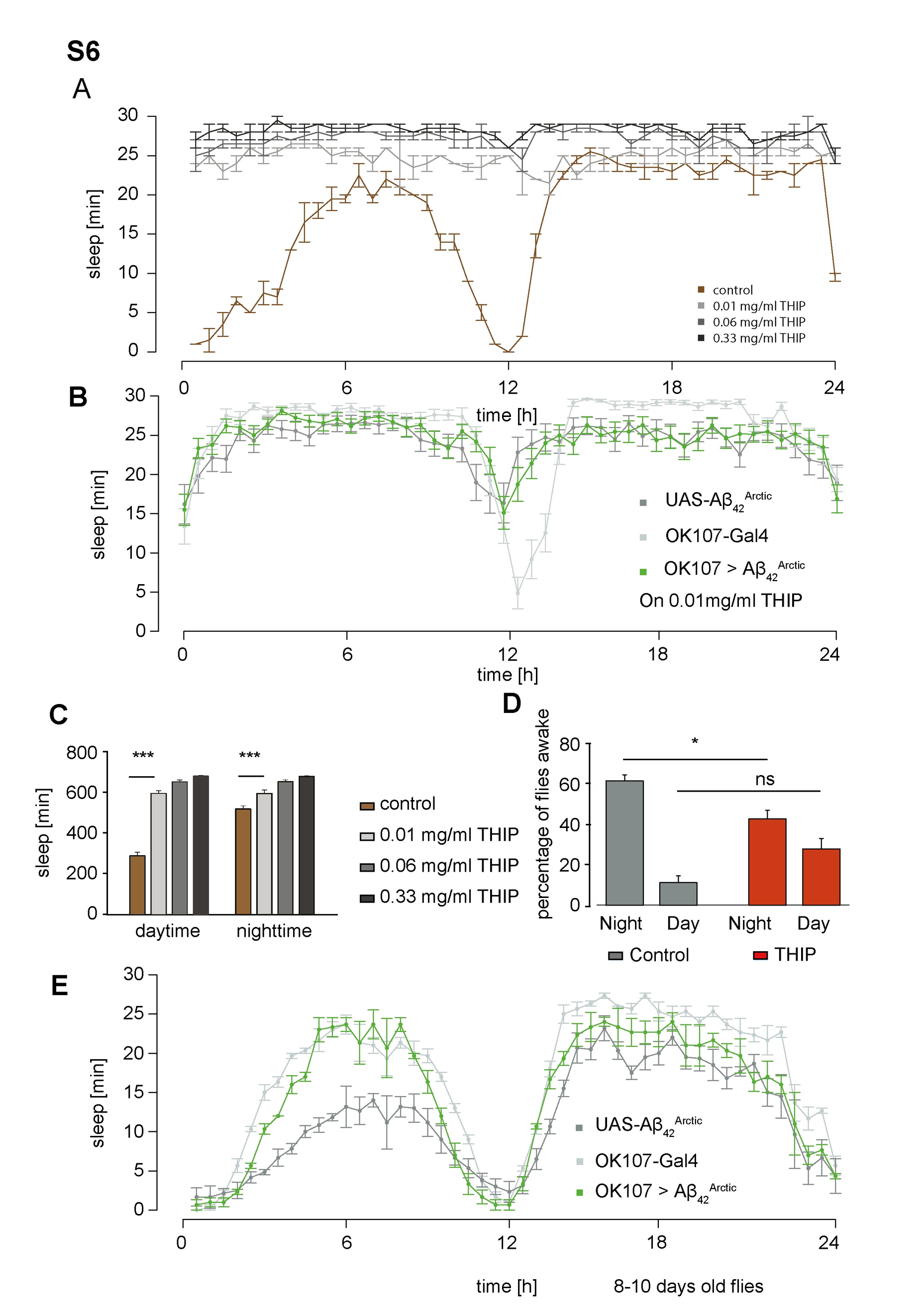

Supplement: S6 Fig — Related to Fig 4. (A) Sleep profile of wild-type Canton-S flies that received different concentrations of THIP or the vehicle. (B) Sleep profile of OK107-Aβ42Arctic flies and parental controls on THIP (C) Quantification of daytime and nighttime sleep; daytime and nighttime sleep was significantly increased in flies that received 0.01, 0.06, or 0.33 mg/ml THIP (n ≥ 24), compared to control flies that received no THIP. (D) Arousal response of THIP-fed flies. At ZT18, a light pulse was used to wake the flies up. (E) Sleep profile of 8- to 10-day-old flies expressing AβArctic in the MB. All other details are similar to Fig 1. MB, mushroom body. (TIF) [file pbio.3001412.s006.tif]

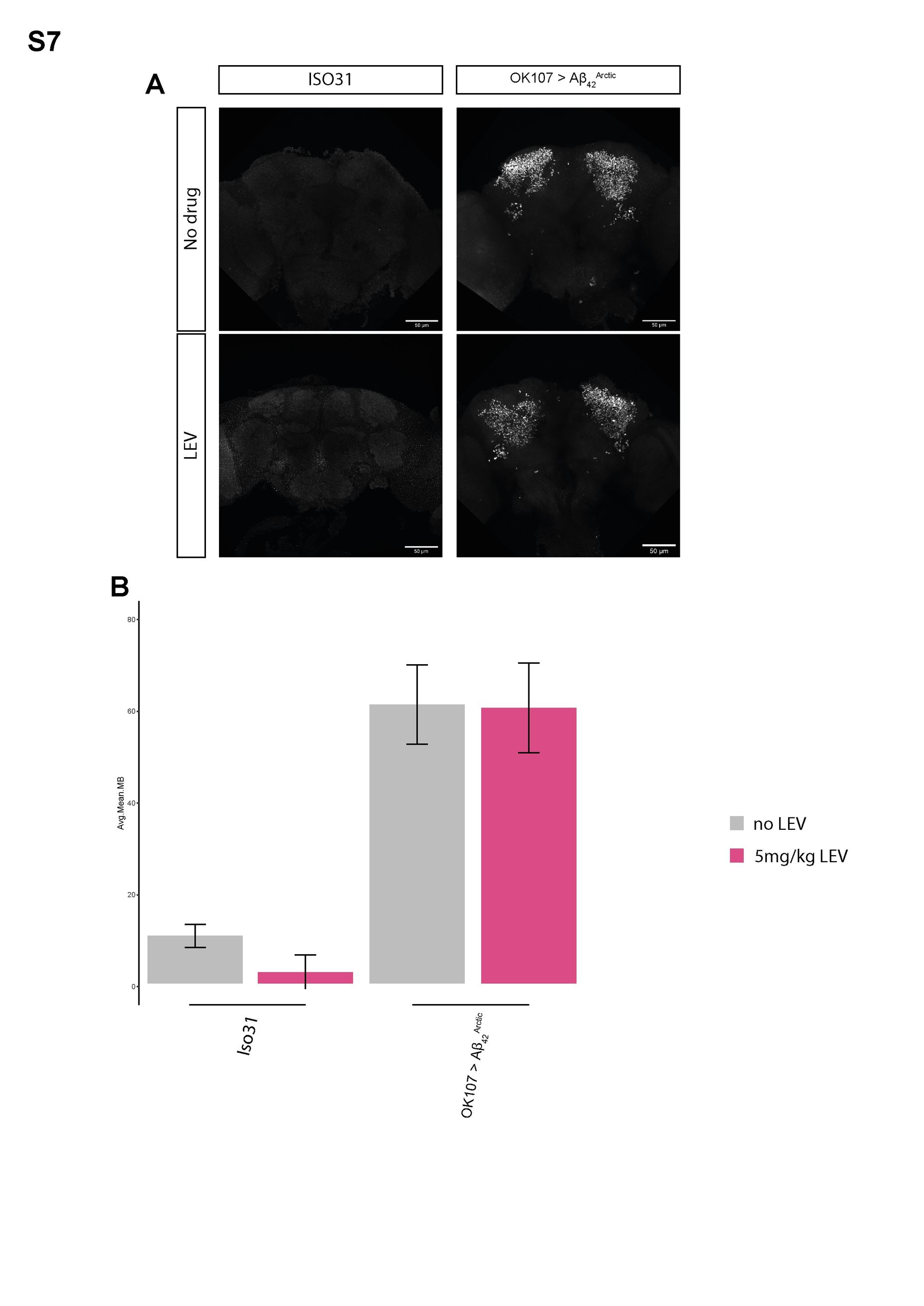

Supplement: S7 Fig — Related to Figs 1 and 3. (A) Whole-mount immunostaining of Iso31 and Ok107>Aβ42Arctic brains with 6E10 antibody. The top row shows untreated flies, and the bottom row brains of flies fed with 5 mg/kg LEV. (B) Quantification of Aβ42Arctic-expression. LEV, Levetiracetam. (TIF) [file pbio.3001412.s007.tif]
